# Decoding the Role of Astrocytes in the Entorhinal Cortex in Alzheimer’s Disease Using High-Dimensional Single-Nucleus RNA Sequencing Data and Next-Generation Knowledge Discovery Methodologies: Focus on Drugs and Natural Product Remedies for Dementia

**DOI:** 10.3389/fphar.2021.720170

**Published:** 2022-02-28

**Authors:** Peter Natesan Pushparaj, Gauthaman Kalamegam, Khalid Hussain Wali Sait, Mahmood Rasool

**Affiliations:** ^1^ Center of Excellence in Genomic Medicine Research, Department of Medical Laboratory Technology, Faculty of Applied Medical Sciences, King Abdulaziz University, Jeddah, Saudi Arabia; ^2^ Center for Transdisciplinary Research, Department of Pharmacology, Saveetha Dental College and Hospitals, Saveetha Institute of Medical and Technical Sciences, Chennai, India; ^3^ Department of Obstetrics and Gynaecology, King Abdulaziz University Hospital, King Abdulaziz University, Jeddah, Saudi Arabia

**Keywords:** astrocytes, alzheimer’s disease and dementia, scREAD, single-nucleus RNA sequencing, in silico tools, anti-rheumatic agents, dasatinib, natural products

## Abstract

**Introduction:** Alzheimer’s disease (AD) is a major cause of the development of cognitive decline and dementia. AD and associated dementias (ADRD) are the major contributors to the enormous burden of morbidity and mortality worldwide. To date, there are no robust therapies to alleviate or cure this debilitating disease. Most drug treatments focus on restoring the normal function of neurons and the cells that cause inflammation, such as microglia in the brain. However, the role of astrocytes, the brain’s housekeeping cells, in the development of AD and the initiation of dementia is still not well understood.

**Objective:** To decipher the role of astrocytes in the entorhinal cortex of AD patients using single nuclear RNA sequencing (snRNASeq) datasets from the Single Cell RNA-seq Database for Alzheimer’s Disease (scREAD). The datasets were originally derived from astrocytes, isolated from the entorhinal cortex of AD brain and healthy brain to decipher disease-specific signaling pathways as well as drugs and natural products that reverse AD-specific signatures in astrocytes.

**Methods:** We used snRNASeq datasets from the scREAD database originally derived from astrocytes isolated from the entorhinal cortex of AD and healthy brains from the Gene Expression Omnibus (GEO) (GSE138852 and GSE147528) and analyzed them using next-generation knowledge discovery (NGKD) platforms. scREAD is a user-friendly open-source interface available at https://bmbls.bmi.osumc.edu/scread/that enables more discovery-oriented strategies. snRNASeq data and metadata can also be visualized and downloaded via an interactive web application at adsn.ddnetbio.com. Differentially expressed genes (DEGs) for each snRNASeq dataset were analyzed using iPathwayGuide to compare and derive disease-specific pathways, gene ontologies, and in silico predictions of drugs and natural products that regulate AD -specific signatures in astrocytes. In addition, DEGs were analyzed using the L1000FWD and L1000CDS2 signature search programming interfaces (APIs) to identify additional drugs and natural products that mimic or reverse AD-specific gene signatures in astrocytes.

**Results:** We found that PI3K/AKT signaling, Wnt signaling, neuroactive ligand-receptor interaction pathways, neurodegeneration pathways, etc. were significantly impaired in astrocytes from the entorhinal cortex of AD patients. Biological processes such as glutamate receptor signaling pathway, regulation of synapse organization, cell-cell adhesion via plasma membrane adhesion molecules, and chylomicrons were negatively enriched in the astrocytes from the entorhinal cortex of AD patients. Gene sets involved in cellular components such as postsynaptic membrane, synaptic membrane, postsynapse, and synapse part were negatively enriched (*p* < 0.01). Moreover, molecular functions such as glutamate receptor activity, neurotransmitter receptor activity, and extracellular ligand-gated ion channels were negatively regulated in the astrocytes of the entorhinal cortex of AD patients (*p* < 0.01). Moreover, the application of NGKD platforms revealed that antirheumatic drugs, vitamin-E, emetine, narciclasine, cephaeline, trichostatin A, withaferin A, dasatinib, etc. can potentially reverse gene signatures associated with AD.

**Conclusions:** The present study highlights an innovative approach to use NGKD platforms to find unique disease-associated signaling pathways and specific synthetic drugs and natural products that can potentially reverse AD and ADRD-associated gene signatures.

## 1 Introduction

Alzheimer’s disease (AD) is a major cause of the development of cognitive decline and dementia in the elderly (Winblad et al., 2016; Matthews et al., 2019). AD-related dementias (ADRD) contribute to 50-70 percent of dementias worldwide (Winblad et al., 2016). AD and associated dementias (ADRD) are the largest contributors to the burden of morbidity and mortality and higher costs in health care systems worldwide (Hurd et al., 2013). Important risk factors for ADRD include ethnicity, age, and gender. Approximately 6.2 million Americans aged 65 years or older were affected by AD and this number is expected to double to 13.8 million by 2060 in the United States of America (United States) (Claxton et al., 2015; Matthews et al., 2019; Alzheimer’s Disease Facts and Figures, 2021). Therefore, ADRD has been declared a health priority worldwide (World Health Organization, 2012). In the United States of America (United States), AD is the sixth leading cause of death in the general population and the fifth leading cause of death in Americans aged 65 years and older. In contrast, reported deaths from other debilitating diseases such as stroke, heart disease, and HIV have declined, while deaths from AD have increased by more than 145% in the U.S. between 2000 and 2019 (Alzheimer’s Disease Facts and Figures, 2021).

**FIGURE 1:**
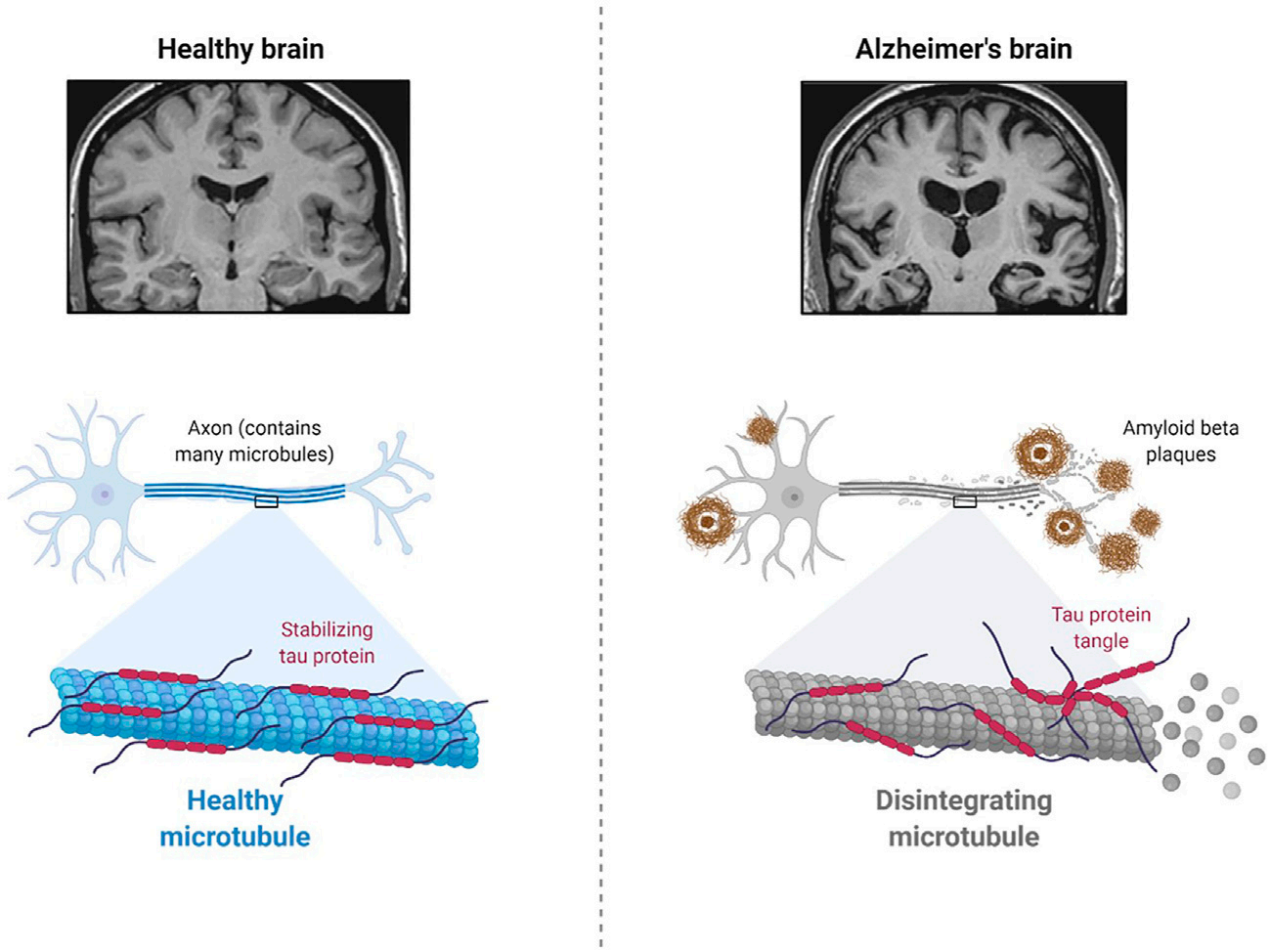
Pathology of Alzheimer’s Disease (Created using Biorender.com).

AD is a neurodegenerative disease of the brain (Figure 1), and symptoms such as cognitive decline and language difficulties have slowly developed in AD patients in recent years. It is mostly diagnosed in the older population with an average age of 65 years or more and is referred to as late-onset AD (LOAD) (Gauthaman et al., 2014; Rasool et al., 2018; Rasool et al., 2021). The progressive damage to neurons from the aggregation of amyloid-beta (Ab) protein and tau protein, as well as neuroinflammation in certain parts of the brain, significantly impairs learning, speech, memory, and other cognitive abilities (Gauthaman et al., 2014; Rasool et al., 2018; Rasool et al., 2021). Importantly, the risk of ADRD is significantly increased in AD patients with diabetes mellitus (Gauthaman et al., 2014; Rasool et al., 2018; Rasool et al., 2021). Moreover, the cellular and molecular mechanisms of AD pathology and the role of specific cells in the brain in the development of ADRD are poorly understood (Rasool et al., 2021).

AD and ADRD pathology differ by brain region, cell type, age, and gender (Sala Frigerio et al., 2019; Rasool et al., 2021). Genome-wide association studies (GWAS) using genetic mapping concepts have revealed genes enriched in AD susceptibility loci, and transcriptomics of whole brain tissue using next-generation sequencing (NGS) platforms or microarray applications have shown an increase in microglial gene connectivity and impairment of neuronal connectivity in AD (Hitzemann et al., 2014). Although transcriptional network dynamics of mass analysis can provide more information about AD pathogenesis, it does not reveal all the dynamic changes at the cellular and molecular levels that contribute to AD pathology. A detailed understanding of the underlying role of individual cell types in AD patients is therefore essential for the development of new therapeutics to treat dementia.

Recent advances in NGS applications such as single-cell RNA sequencing (scRNA-Seq) have enabled researchers to study and understand the dynamic transcriptomic profile of individual cells in brain tissue or other biological samples. RNA-sequencing of posterior cingulate astrocytes (PC) in AD patients revealed differential expression of mitochondria-related genes, including TRMT61B, FASTKD2, and NDUFA4L2. In addition, immune response genes such as CLU, C3, and CD74 were identified to play a central role in the generation or clearance of amyloid-beta (Sekar et al., 2015). scRNASeq provides a higher resolution of cellular dynamics and a better understanding of individual cells in the tissue microenvironment (Grubman et al., 2019; Jiang et al., 2020; Wu and Zhang, 2020). Similarly, the single nucleus RNA sequencing (snRNA-Seq) technique is used to study frozen samples where dissociation of single cells becomes a problem and affects gene expression patterns. Although AD is one of the major reasons for the development of cognitive decline and dementia (Gauthaman et al., 2014; Rasool et al., 2018; Rasool et al., 2021), there are still no robust therapies to alleviate or cure this debilitating disease (Gao et al., 2016; Rasool et al., 2018) and most drug treatments focus on restoring normal function of cells that cause inflammation, such as microglia and neurons in the brain (Oksanen et al., 2017). However, the genetic basis of astrocytes in the development of AD, and the triggering of dementia is still not clearly understood (Oksanen et al., 2017; Kery et al., 2020). Therefore, a precise understanding of the underlying role of astrocytes in AD patients may provide clues for the development of effective therapies to treat dementia. Here, we used an innovative approach to leverage next-generation knowledge discovery (NGKD) platforms to decipher the AD -specific gene signatures in astrocytes isolated from the entorhinal cortex of AD patients and specific synthetic drugs and natural products to improve AD and associated disease pathologies such as dementia.

## 2 Materials and Methods

### 2.1 Ethical Statement

This study was exempt from Institutional Review Board (IRB) approval because it did not involve animal models or human subjects. It was performed using DEGs derived from the Single Cell RNA-seq Database for Alzheimer’s Disease (scREAD) based on publicly available and previously published single nucleus RNA sequencing datasets from the Gene Expression Omnibus (GEO).

### 2.2 Data Source

In the present study, we snRNASeq data from the scREAD, originally obtained from astrocytes isolated from the entorhinal cortex of AD brains and healthy brains from the Gene Expression Omnibus (GEO) (GSE138852 and GSE147528). scREAD is a user-friendly open-source interface available at https://bmbls.bmi.osumc.edu/scread/to enable more discovery-oriented strategies (Wu and Zhang, 2020; Jiang et al., 2020; Jiang et al., 2021) (Supplementary Figure S1). Datasets were filtered in scREAD by selecting the options for species (human), condition (all), region in the brain (entorhinal cortex), and gender (all), and are listed in Table 1 with the corresponding Braak levels (Braak and Braak, 1991). scREAD webtool was also used to visualize all cell types and sub-clusters of astrocytes in the entorhinal cortex region of the brain using Uniform Manifold Approximation and Projection (UMAP) (Becht et al., 2018). All snRNASeq data are freely available in the Gene Expression Omnibus (GEO) under accession numbers GSE138852 and GSE147528.

**TABLE 1:** Information on the snRNASeq datasets obtained from scREAD database for NGKD analysis (Human)*.

scREAD Data ID	File name	Condition	Brain region	Sex	Braak Stage	GEO ID	Number of cells
AD00201	H-H-Entorhinal Cortex-Male	Control	Entorhinal cortex	Male	NA	GSE138852 (*n* = 6); GSE147528 (*n* = 3)	29,993
AD00202	H-H-Entorhinal Cortex-Female	Control	Entorhinal cortex	Female	NA	GSE138852 (*n* = 2)	1,122
AD00203	H-AD-Entorhinal Cortex-Male_001	Disease	Entorhinal cortex	Male	4–5	GSE138852 (*n* = 6)	3,770
AD00204	H-AD-Entorhinal Cortex-Female_001	Disease	Entorhinal cortex	Female	4	GSE138852 (*n* = 2)	2,303
AD00205	H-AD.Braak 2-Entorhinal cortex -Male_001	Disease	Entorhinal cortex	Male	2	GSE147528 (*n* = 3)	25,492
AD00206	H-AD.Braak 6-Entorhinal cortex -Male_001	Disease	Entorhinal cortex	Male	6	GSE147528 (*n* = 3)	25,537

NA, not applicable; the mean age range of samples from the GSE138852 dataset was 77.6 (range 67.3–91 years) and the mean age range of samples from the GSE147528 dataset was 74.4 (range, 50–91 years).

Importantly, the snRNAseq datasets (GSE138852) are available via an interactive web application at adsn.ddnetbio.com (Grubman et al., 2019). The characteristics of all AD and healthy snRNASeq scREAD datasets used in this study are provided in Table 1. As of May 2021, the snRNASeq datasets used for this study had been already published and are publicly available (Barrett et al., 2013).

### 2.3 The snRNASeq Data Analysis Using iPathwayGuide

**FIGURE 2:**
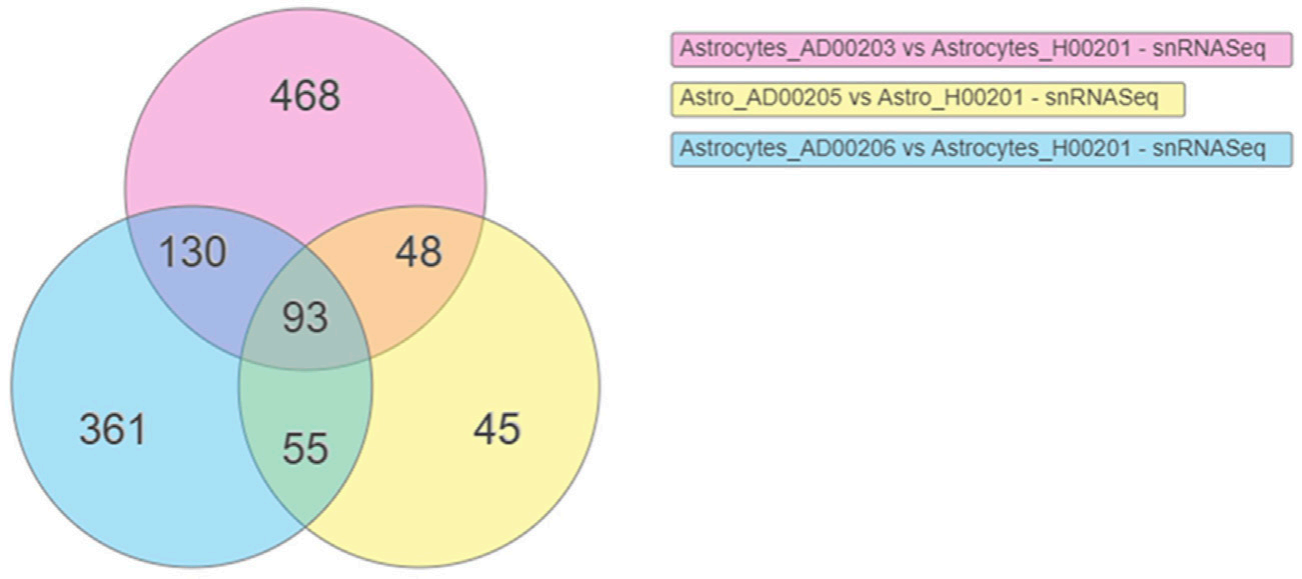
Venn diagram showing the DEGs obtained using the scREAD analysis of snRNASeq data from astrocytes of AD groups (AD00203, AD00205, and AD00206) compared to astrocytes of the healthy control group (AD00201) were 739, 241, and 639 respectively after filtering with a *p*-value cut off of 0.05 and log2 fold change (Log2Fc) of ±0.3. Further analysis of DEGs using iPathwayGuide software showed that 93 DEGs were commonly regulated in all the disease groups.

DEGs were obtained using scREAD analysis of snRNASeq data from astrocytes of AD groups (AD00203, AD00205, and AD00206) compared with the healthy control group (AD00201). DEGs of AD groups (AD00203, AD00205, and AD00206) were further filtered using a *p*-value cut-off of 0.05, and log2 fold change (Log2Fc) of ±0.3 in iPathwayGuide Software (Advaita Bioinformatics, United States) to obtain 739, 241, and 639 DEGs. Further analysis of these DEGs using iPathwayGuide software showed that 93 DEGs were commonly regulated in all disease groups (Figure 2). The Kyoto Encyclopedia of Genes and Genomes (KEGG) database was used to decipher differentially regulated pathways (Kanehisa and Goto, 2000; Kanehisa et al., 2002; Kanehisa et al., 2010; Kanehisa et al., 2012; Kanehisa et al., 2014), and the Gene Ontology Consortium database (Ashburner et al., 2000; Gene Ontology Consortium 2001) was used to identify the differentially regulated GO functions, and the Comparative Toxicogenomics Database was used to find the chemicals/drugs/toxicants (CDT and the KEGG database for diseases (Kanehisa and Goto, 2000; Kanehisa et al., 2002). The iPathwayGuide software used the Impact Analysis Method (IAM) (Draghici et al., 2003; Draghici et al., 2007; Draghici, 2011) to obtain significantly impacted DEGs and pathways compared with the corresponding control group; the *p*-value computed using Fisher’s method was used to determine the pathway score, and the *p*-value was adjusted based on the false discovery rate (FDR) (Benjamini and Hochberg, 1995; Benjamini and Yekutieli, 2001). and Bonferroni multiple testing corrections (Bonferroni, 1935). The *p*-values were computed based on the hypergeometric distribution in iPathwayGuide analysis and the FDR and Bonferroni methods for multiple testing corrections (Draghici et al., 2003; Draghici, 2011).

### 2.4 Determination of Upstream Drugs and Natural Products Using iPathwayGuide

The determination of upstream drugs or natural products was predicted based on the enrichment of DEGs and 2) a network of connections or interactions from the Advaita Knowledge Base (Draghici et al., 2003; Draghici, 2011). The iPathwayGuide analysis was based on two hypotheses (HP and HA). The overly abundant or present upstream chemical, drug, or toxicant (CDT) was predicted under the conditions analyzed under the first hypothesis called HP and the upstream CDT. is insufficient (or absent) was predicted under the conditions analyzed under the second hypothesis. HA. iPathwayGuide calculates a Z-score for each CDT z(u) by iterating over the genes in DT(u) and their incoming edges in (g) in testing both HP and HA. Subsequently, the *p*-value was computed corresponding to the z-score Pz (One-Tailed) under the probability density function for a normal distribution, N (0,1) (Draghici et al., 2003; Draghici, 2011).

#### 2.4.1 Determination of Upstream Drugs and Natural Products Present or Overly Abundant Using iPathwayGuide

To determine the presence or abundance of CDTs based on the differentially expressed (DE) genes, CDT u, DE genes downstream of u, DTA (u) were compared to measured target genes predicted by chance to be both DE and consistent. An over-representation method was applied to calculate the statistical significance (*p*-value) based on the number of consistent DE genes in the iPathwayGuide analysis. The Ppres (*p*-value) was calculated based on the hypergeometric distribution (Draghici et al., 2003; Draghici, 2011). Then, the global probability value (PG) was computed by combining Pz and Ppres: and was used to rank the upstream regulators and test the HP research hypothesis. The *p*-values were combined into one test statistic using the standard Fisher’s method.

#### 2.4.2 Determination of Upstream Drugs and Natural Products Absent or Insufficient Using iPathwayGuide

To determine the absence or insufficiency of CDTs based on the DE genes, Pabs was calculated using the iPathwayGuide analysis. The upstream CDTs that were absent or insufficient under the conditions investigated based on the number of consistent DE genes downstream of u, and DTI (u) was compared to the measured target genes predicted by chance to be both DE and consistent. The Pabs (*p*-value) was calculated based on the hypergeometric distribution (Draghici et al., 2003; Draghici, 2011). Then the PG was computed by combining Pz and Pabs and was used to rank the upstream regulators that were absent or insufficient and to test the research hypothesis HA. The analysis combines Pabs and Pz, using Fisher’s method as described previously, where Pz was measured only for significant negative z-scores (z ≤ −2) (Draghici et al., 2003; Draghici, 2011).

### 2.5 L1000FWD and L1000CDS^2^ Analyses

DEGs were subjected to L1000 Fire Works Display (L1000FWD) analysis using the L1000FWD signature search application programming interface (API) (Wang et al., 2018) to identify the top 50 drugs and natural products that have the potential to reverse AD-associated signaling. Similarly, the same set of DEGs was subjected to L1000 Characteristic Direction Signature Search Engine (L1000CDS2) analysis using the L1000CDS2 Signature Search API to identify the top 50 drugs and natural products with the potential to reverse AD-associated signaling (Duan et al., 2016).

## 3 Results

In the present study, snRNASeq datasets of astrocytes isolated from the entorhinal cortex region of AD patients and healthy brains were obtained from the scREAD database for NGKD platform analysis (Table 1). The scREAD web tool was used to visualize all cell types and sub-clusters of astrocytes in the entorhinal cortex region of the brain in AD and healthy snRNASeq datasets using UMAP (Supplementary Figure S1). A UMAP example for the healthy control and AD scREAD datasets is shown in Supplementary Figure S2.

**FIGURE 3:**
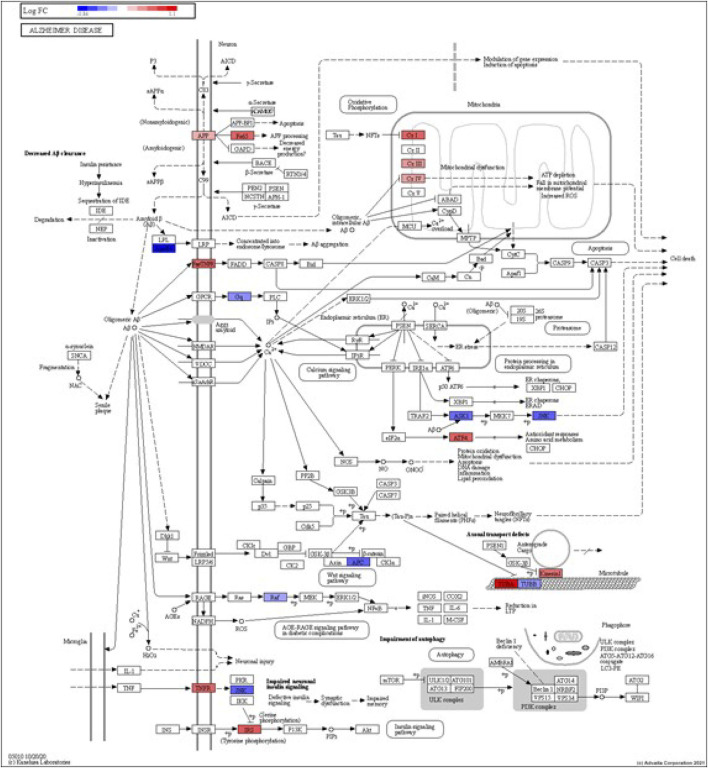
iPathwayGuide analysis shows the differentially regulated genes in the KEGG Alzheimer’s disease pathway in astrocytes from the AD group (AD00203) compared to astrocytes from the healthy control group (AD00201).

**FIGURE 4:**
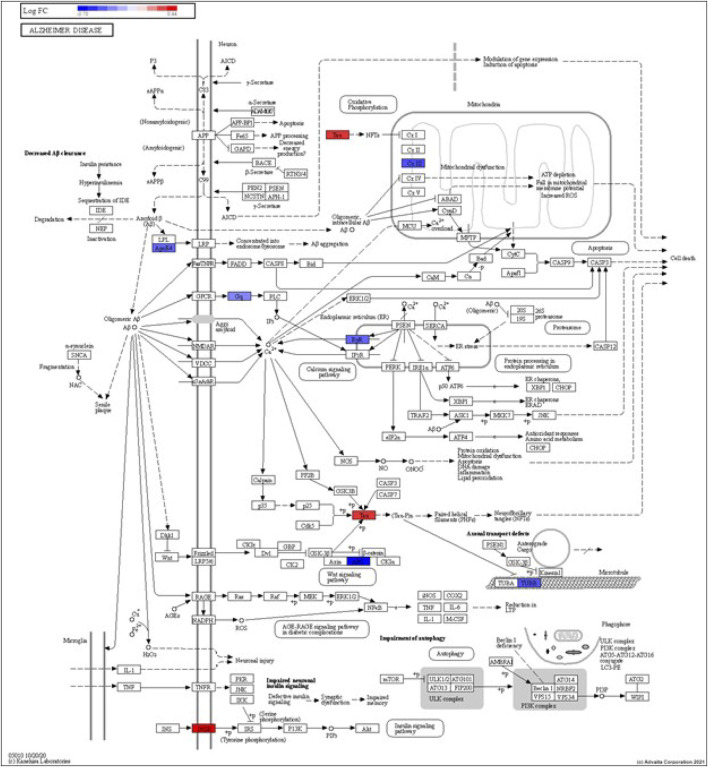
iPathwayGuide analysis shows the differentially regulated genes in the KEGG Alzheimer’s disease pathway in astrocytes from the AD group (AD00205) compared to astrocytes from the healthy control group (AD00201).

**FIGURE 5:**
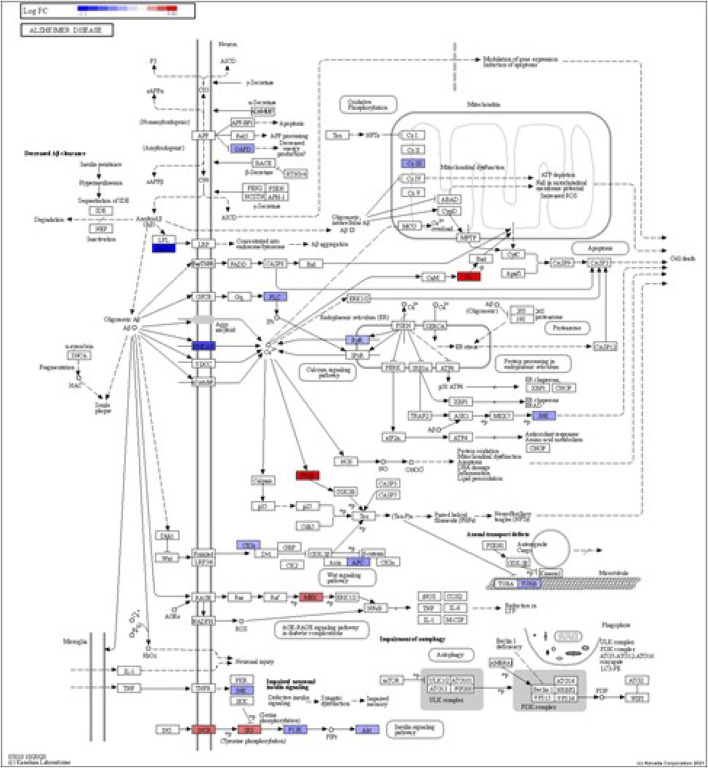
iPathwayGuide analysis shows the differentially regulated genes in the KEGG Alzheimer’s disease pathway in astrocytes from the AD group (AD00206) compared to astrocytes from the healthy control group (AD00201).

**FIGURE 6:**
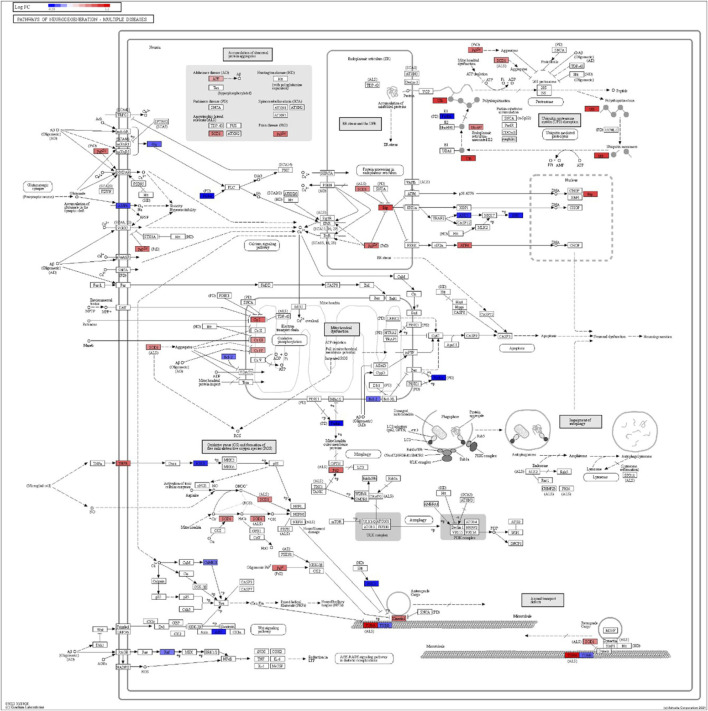
iPathwayGuide analysis shows the differentially regulated genes in the KEGG neurodegeneration pathway (multiple diseases) in the astrocytes of the AD group (AD00203) compared to the astrocytes of the healthy control group (AD00201).

**FIGURE 7:**
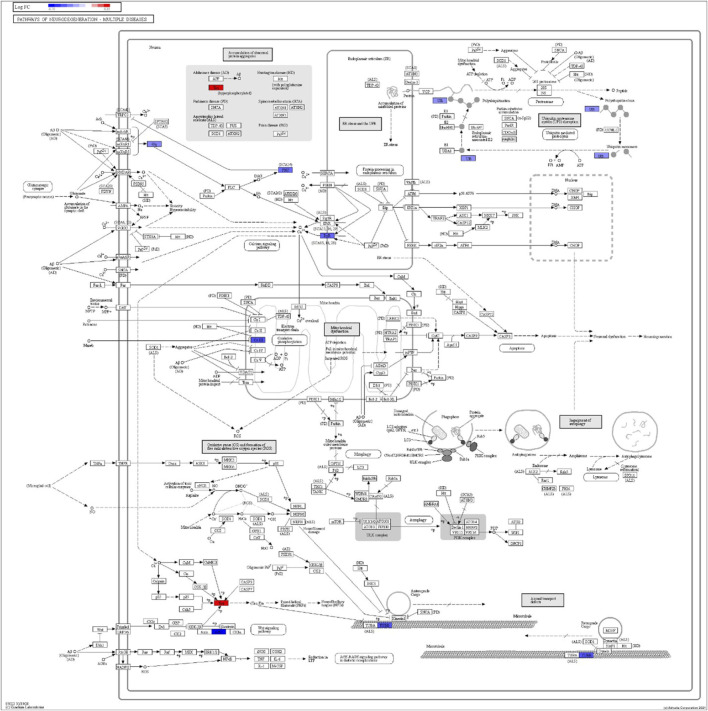
iPathwayGuide analysis shows the differentially regulated genes in the KEGG neurodegeneration pathway (multiple diseases) in the astrocytes of the AD group (AD00205) compared to the astrocytes of the healthy control group (AD00201).

**FIGURE 8:**
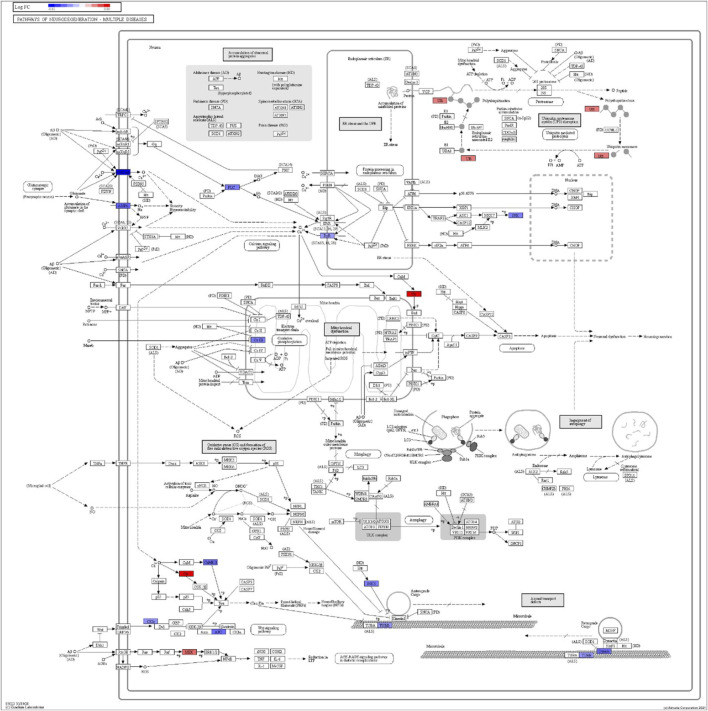
iPathwayGuide analysis shows the differentially regulated genes in the KEGG neurodegeneration pathway (multiple diseases) in the astrocytes of the AD group (AD00206) compared to the astrocytes of the healthy control group (AD00201).

DEGs in astrocytes from the entorhinal cortex compared to healthy controls were determined using paired comparisons with the healthy control (AD00201) and AD (AD00203, AD00205, and AD00206) datasets (Supplementary Tables S1–S3) The 93 DEGs common to all AD datasets can be found in Supplementary Table S4. The 15 pathways most affected by DEGs in the AD groups compared to healthy controls are listed in Tables 2–4. Based on the number of DEGs, the top signaling pathways differentially regulated in the astrocytes of AD patients in the context of neurodegeneration include Alzheimer’s disease, prion disease, Parkinson’s disease, Huntington’s disease, neurodegeneration signaling pathways (multiple diseases), amyotrophic lateral sclerosis, and the phosphatidylinositol 3-kinases/protein kinase B (PI3K/AKT) pathway. The differentially regulated KEGG pathways of Alzheimer’s disease in the groups of AD are shown in Figures 3–5, and the differentially regulated KEGG pathways of neurodegenerative degeneration (multiple diseases) are shown in Figures 6–8. Analysis of WNT pathway perturbation and PI3K/AKT pathways followed by iPathwayGuide coherent cascade activation revealed the dysregulation of these pathways in the astrocytes of AD patients from the entorhinal cortex (Supplementary Figure S3A and Supplementary Figure S4B). The differentially regulated genes in the WNT pathways and the PI3K/AKT pathways are also shown in Supplementary Figure S3B and Supplementary Figure S4B, respectively. In addition to the neurodegenerative diseases, we also observed the signaling pathways associated with *Salmonella* infection, human papillomavirus (HPV) infection, and human T-cell leukemia virus infection in the astrocytes of the severe AD groups (Table 2 and Table 4) compared with the healthy controls. Gene set enrichment analysis (GSEA) showed that gene sets involved in cellular components, such as postsynaptic membrane, synaptic membrane, postsynapse, synapse, and synapse, were negatively enriched (*p* < 0.01). Neuroactive ligand-receptor interaction based on KEGG pathways was significantly downregulated (*p* < 0.01), and cellular function of the transporter complex was also negatively enriched (*p* < 0.01). Similarly, genes associated with glutamate receptor activity, neurotransmitter receptor activity, glutamate receptor signaling, heterophilic cell-cell adhesion via plasma membrane cell adhesion molecules, cell-cell adhesion via plasma membrane adhesion molecules, and behavior were also negatively enriched (*p* < 0.01) in astrocytes from AD patients (Table 5). Importantly, differential expression of GWAS genes in astrocytes from the entorhinal cortex in the brain of AD is listed in Table 6. The most downregulated GWAS genes in astrocytes from the entorhinal cortex associated with the pathogenesis of AD were NKAIN3, LRRC4C, CADM2, DLC1, APOE, TNIK, GADD45G, FRMD4A, CTNNA2, NPAS3, NCKAP5, and RORA.

**TABLE 2:** Top 15 pathways ranked based on their associated differentially expressed genes derived from astrocytes based on the comparison AD00203 (disease) vs. AD00201 (control).

pName	countDE	countAll	pv	pAcc	pComb	pORA
Metabolic pathways	66	74	0.646507	—	—	0.57601
Pathways of neurodegeneration- multiple diseases	32	33	0.065081	0.114443	0.065081	0.10487
Pathways in cancer	26	29	0.402445	0.22089	0.402445	0.604649
Protein processing in endoplasmic reticulum	25	25	0.041699	0.133433	0.041699	0.05241
Amyotrophic lateral sclerosis	25	26	0.274844	0.384808	0.274844	0.200526
*Salmonella* infection	23	24	0.024458	0.015492	0.024458	0.239293
MAPK signaling pathway	22	25	0.023567	0.004998	0.023567	0.710082
Prion disease	22	22	0.248451	0.894553	0.248451	0.075041
Huntington disease	22	22	0.268695	0.996502	0.268695	0.075041
Alzheimer disease	22	23	0.493084	0.69965	0.493084	0.260971
Parkinson disease	21	21	0.194912	0.572214	0.194912	0.084551
PI3K-Akt signaling pathway	20	23	0.022861	0.004498	0.022861	0.761224
Shigellosis	18	18	0.346957	0.888556	0.346957	0.120833
Human papillomavirus infection	18	19	0.66544	0.832584	0.66544	0.364583
Non-alcoholic fatty liver disease	16	16	0.239509	0.416792	0.239509	0.153188

**TABLE 3:** Top 15 pathways ranked based on their associated differentially expressed genes derived from astrocytes based on the comparison AD00205 (disease) vs. AD00201 (control).

pName	countDE	countAll	pv	pAcc	pComb	pORA
Metabolic pathways	26	39	0.681028	—	—	0.633724
MAPK signaling pathway	10	12	0.050732	0.044978	0.050732	0.196978
Pathways in cancer	10	14	0.694137	0.646677	0.694137	0.507905
PI3K-Akt signaling pathway	9	11	0.308503	0.358821	0.308503	0.252895
Cell adhesion molecules	8	8	0.103009	—	—	0.042457
Pathways of neurodegeneration—multiple diseases	8	15	0.881576	0.596702	0.881576	0.929541
Alzheimer disease	8	15	0.913995	0.661669	0.913995	0.929541
Morphine addiction	7	7	0.188261	0.729635	0.188261	0.063322
Calcium signaling pathway	7	8	0.307444	0.431284	0.307444	0.209381
Mineral absorption	7	8	0.340951	—	—	0.209381
Axon guidance	7	10	0.878371	0.934533	0.878371	0.587743
Prion disease	7	13	0.923744	0.695152	0.923744	0.914648
Hippo signaling pathway	6	6	0.110827	0.246877	0.110827	0.09431
Purine metabolism	6	6	0.191181	—	—	0.09431
Phospholipase D signaling pathway	6	7	0.398858	0.470265	0.398858	0.280235

**TABLE 4:** Top 15 pathways ranked based on their associated differentially expressed genes derived from astrocytes based on the comparison AD00206 (disease) vs. AD00201 (control).

pName	countDE	countAll	pv	pAcc	pComb	pORA
Metabolic pathways	56	65	0.305591	—	—	0.179067
Pathways in cancer	27	33	0.873799	0.96052	0.873799	0.563988
MAPK signaling pathway	22	27	0.588314	0.409295	0.588314	0.596373
PI3K-Akt signaling pathway	20	21	0.188374	0.663668	0.188374	0.069671
Axon guidance	20	23	0.69189	0.96052	0.69189	0.339855
Pathogenic *Escherichia coli* infection	18	21	0.361799	0.273863	0.361799	0.416672
cAMP signaling pathway	18	23	0.547391	0.290855	0.547391	0.743484
Alzheimer disease	18	24	0.631722	0.324838	0.631722	0.850642
Pathways of neurodegeneration - multiple diseases	17	23	0.927345	0.735632	0.927345	0.875279
Ribosome	16	16	0.085705	—	—	0.033724
Human T-cell leukemia virus 1 infection	15	20	0.164459	0.045977	0.164459	0.841122
*Salmonella* infection	15	22	0.722322	0.369315	0.722322	0.960377
Regulation of actin cytoskeleton	15	19	0.882177	0.773113	0.882177	0.718756
Osteoclast differentiation	15	19	0.886352	0.783108	0.886352	0.718756
Rap1 signaling pathway	14	17	0.283435	0.135432	0.283435	0.594732

**TABLE 5:** Top 25 Impacted Pathways obtained using Gene Set Enrichment Analysis (GSEA) based on Normalized Enrichment Score (NES) and False Discovery Rate (FDR) using the web tool available at http://adsn.ddnetbio.com/

BP/CC/MF/KEGG	Impacted pathway	NES	FDR
CC	Postsynaptic membrane	−2.02	0.001283
CC	Synaptic membrane	−1.858	0.001283
CC	Postsynapse	−1.653	0.001283
CC	Synapse	−1.484	0.001283
CC	Synapse part	−1.47	0.001997
KEGG	Neuroactive ligand receptor interaction	−1.98	0.004235
MF	Glutamate receptor activity	−2.169	0.005663
MF	Extracellular ligand gated ion channel activity	−2.11	0.005663
CC	Transporter complex	−1.685	0.006176
MF	Neurotransmitter receptor activity	−2.081	0.009856
BP	Glutamate receptor signaling pathway	−2.206	0.011
BP	Regulation of synapse organization	−1.985	0.011
BP	Single organism behavior	−1.744	0.011
BP	Behavior	−1.675	0.011
BP	Heterophilic cell-cell adhesion via plasma membrane cell adhesion molecules	−2.09	0.01224
BP	Regulation of synaptic transmission glutamatergic	−2.03	0.01224
BP	Synaptic signaling	−1.682	0.01224
CC	Chylomicron	−1.639	0.01265
BP	Cell-cell adhesion *via* plasma membrane adhesion molecules	−1.895	0.01373
BP	Synapse organization	−1.876	0.01619
BP	Learning	−1.831	0.01867
MF	Extracellular glutamate gated ion channel activity	−2.007	0.01954
MF	Ligand-gated channel activity	−1.83	0.02148
CC	Plasma membrane region	−1.353	0.02471
BP	Startle response	−2.044	0.02524

**TABLE 6:** Top 25 differentially expressed GWAS genes in astrocytes from entorhinal cortex in AD brain based on analysis using the web tool available at http://adsn.ddnetbio.com/.

Gene Name	LogFc	FDR	Category
NKAIN3	−1.965	8.226e-103	Biomarkers
LRRC4C	−1.54	1.055e-51	Biomarkers
CADM2	−0.9309	3.203e-43	Alzheimer’s
DLC1	−1.269	2.297e-38	Alzheimer’s | LOAD
APOE	−1.136	5.424e-35	Alzheimer’s | Biomarkers | LOAD
TNIK	−0.8955	3.230e-34	Biomarkers
GADD45G	1.232	1.193e-33	Biomarkers
FRMD4A	−1.263	1.728e-28	Alzheimer’s
CTNNA2	−0.7049	5.649e-28	Alzheimer’s | Biomarkers
NPAS3	−0.5062	6.715e-28	Biomarkers
NCKAP5	−1.027	9.935e-26	Alzheimer’s
RORA	−0.6096	4.061e-25	Alzheimer’s | Biomarkers
FBXL7	−0.8531	7.555e-24	Alzheimer’s
AHNAK	1.015	1.846e-23	Alzheimer’s | Biomarkers | LOAD
FAT3	−1.066	4.009e-22	Alzheimer’s | Biomarkers | LOAD
SLCO3A1	0.9594	5.436e-19	Biomarkers | Neuropathologic
SH3RF1	−1.016	5.596e-17	Alzheimer’s
CACNA2D3	0.8438	2.955e-16	Alzheimer’s
DLG2	−0.6662	1.094e-15	Biomarkers
PDE7B	−0.7986	8.458e-14	Alzheimer’s
SPON1	−0.7795	1.476e-13	Alzheimer’s
PTPRG	−0.764	2.860e-13	Alzheimer’s
CDH23	0.785	3.357e-12	Biomarkers
AUTS2	−0.5907	2.649e-11	Biomarkers
LUZP2	−0.8184	7.842e-11	Alzheimer’s | Biomarkers

Comparative analysis of the AD datasets from the scREAD based on the DEGs with iPathwayGuide showed that the antirheumatic drugs, vitamin E, salinomycin, and clorgyline have insufficient (*p* < 0.05) signaling effect in the astrocytes of AD patients (Table 7). In addition, Tables 8–10 list the drugs or natural products that could potentially reverse the gene signatures of astrocytes in the AD groups (AD00203, AD00205, and AD00206) based on the L1000FWD web tool analysis. L1000FWD analysis revealed that natural products such as emetine, cephaeline, homoharringtonine, narciclasine, withaferin A and several synthetic drugs such as dasatinib can significantly reverse gene signatures associated with AD pathology.

**TABLE 7:** The upstream Chemicals, Drugs, or Toxicants (CDTs) were predicted as absent (or insufficient) in the astrocytes of AD based on the number of DEGs significantly impacted in each category.

Name	cDE_n (Astrocytes_AD00203 vs. Astrocytes_AD00201)	cDE (Astrocytes_AD00203 vs. Astrocytes_AD00201)	pv_comb_n (Astrocytes_AD00203 vs. Astrocytes_AD00201)	cDE_n (Astro_AD00205 vs. Astro_AD00201)	cDE (Astro_AD00205 vs. Astro_H00201)	pv_comb_n (Astro_AD00205 vs. Astro_AD00201)	cDE_n (Astrocytes_AD00206 vs. Astrocytes_AD00201)	cDE (Astrocytes_AD00206 vs. Astrocytes_AD00201)	pv_comb_n (Astrocytes_AD00206 vs. Astrocytes_AD00201)
Antirheumatic Agents	116	193	0.017467	36	67	1	131	189	9.71E-07
perfluorooctanoic acid	43	64	0.029342	13	17	0.906879	23	40	0.99999999
Vitamin E	40	110	1	10	31	1	50	76	0.0281796
Vanadates	35	65	1	15	19	0.035627	51	76	0.01079637
Piroxicam	28	34	0.001769	6	11	0.988182	13	21	0.99599971
Propionaldehyde	28	61	1	17	29	0.999354	46	68	0.01320547
MT19c compound	19	25	0.029523	3	7	0.943716	4	18	1
Uranium Compounds	16	19	0.010291	1	4	0.999381	9	10	0.02177297
methylselenic acid	15	30	1	9	12	0.900238	20	25	0.03745406
nickel chloride	15	32	1	9	11	0.048969	8	30	1
3-(4′-hydroxy-3′-adamantylbiphenyl-4-yl)acrylic acid	15	16	0.000817	3	6	0.988999	11	12	0.00697841
CD 437	14	15	0.002889	2	6	0.998675	11	15	0.89309347
Salinomycin	11	13	0.034563		4	1	10	18	0.99353861
cylindrospermopsin	10	17	0.999219	8	9	0.034748	5	15	0.99999998
Clorgyline	10	11	0.021724	5	5	0.039888	8	9	0.0461479
Aldehydes	10	23	1	11	14	0.045315	22	28	0.00958826
Environmental Pollutants	8	8	0.020578	1	1	0.646199	2	2	0.61565284
chloroacetaldehyde	7	15	0.999971	2	3	0.871198	11	13	0.03735946
Dinitrochlorobenzene	7	14	0.999971	9	10	0.003928	6	16	0.99998432
cadmium sulfate	5	20	1	7	8	0.018781	3	10	0.99,987,959
Sulforafan	3	11	1	6	6	0.020331	6	12	0.9998163
Bezafibrate	2	3	0.94972	1	1	0.875495	4	4	0.04,955,141

**TABLE 8:** The top 50 drugs or natural products that reverse DEGs of astrocytes from entorhinal cortex in AD (AD00203 (disease) vs AD00201 (control) based on L1000FWD analysis.

Signature ID	Drug	Similarity score	*p*-value	q-value	Z-score	Combined score
CPC006_HEPG2_6H:BRD-K01976263-003-04-5:0.63	Emetine	−0.0673	1.16E-10	7.11E-07	1.79	−17.83
CPC017_MCF7_24H:BRD-A62184259-001-02-8:10	Cycloheximide	−0.0617	6.64E-10	3.28E-06	1.72	−15.78
HOG003_A549_24H:BRD-K01976263-003-04-5:3.3333	Emetine	−0.0598	7.55E-09	2.31E-05	1.62	−13.18
CVD001_HEPG2_24H:BRD-K03067624-001-01-5:10	Emetine	−0.0579	1.59E-08	4.25E-05	1.69	−13.16
CPC006_HT29_6H:BRD-K01976263-003-04-5:0.63	Emetine	−0.0561	3.40E-07	3.41E-04	1.77	−11.45
CPC014_SKB_24H:BRD-M16762496-001-01-9:10	PIK-75	−0.0542	8.37E-07	5.60E-04	1.69	−10.25
CPC004_HCC515_24H:BRD-A25687296-300-03-5:10	Emetine	−0.0523	1.60E-06	8.80E-04	1.83	−10.6
CPC018_MCF7_24H:BRD-K36055864-001-09-3:10	Cycloheximide	−0.0523	6.02E-07	4.47E-04	1.71	−10.61
CPC002_HCC515_24H:BRD-K80348542-001-01-4:10	Cephaeline	−0.0505	7.75E-06	2.67E-03	1.8	−9.2
CPC008_A375_24H:BRD-K66032149-001-01-9:10	VU-0365117-1	−0.0486	2.18E-05	5.87E-03	1.74	−8.12
CPC017_HEPG2_6H:BRD-A25687296-300-03-5:10	Emetine	−0.0486	3.66E-06	1.61E-03	1.7	−9.22
CPC004_HT29_6H:BRD-A25687296-300-03-5:10	Emetine	−0.0467	2.11E-05	5.82E-03	1.82	−8.51
CPC009_PC3_6H:BRD-K21773564-001-01-8:10	BRD-K21773564	−0.0467	2.26E-05	6.04E-03	1.73	−8.05
CPC004_HEPG2_6H:BRD-A62184259-001-02-8:10	Cycloheximide	−0.0467	1.10E-05	3.52E-03	1.85	−9.2
CPC006_HEPG2_6H:BRD-A45889380-300-04-8:10	Mepacrine	−0.0467	1.28E-05	3.96E-03	1.84	−8.99
CPC016_HEPG2_6H:BRD-K80348542-001-01-4:10	Cephaeline	−0.0449	2.86E-05	7.24E-03	1.74	−7.92
CPC017_A549_24H:BRD-K11927976-050-01-1:10	ER-27319	−0.0449	4.63E-05	9.86E-03	1.72	−7.44
CPC013_SKB_24H:BRD-K87909389-001-01-2:10	Alvocidib	−0.0449	3.78E-05	8.51E-03	1.72	−7.62
CPC018_A549_6H:BRD-K63606607-001-01-8:10	Bufalin	−0.0449	3.07E-05	7.68E-03	1.72	−7.74
CPC004_VCAP_24H:BRD-A01593789-001-02-3:10	Chlormadinone	−0.0449	1.07E-04	1.71E-02	1.81	−7.19
CPC004_HA1E_6H:BRD-K14920963-304-01-9:10	Erythrosine	−0.0449	8.86E-05	1.49E-02	1.83	-7.42
CVD001_HUH7_6H:BRD-K03067624-001-01-5:10	Emetine	−0.0449	1.03E-04	1.68E-02	1.65	−6.59
CPC017_MCF7_6H:BRD-A25687296-300-03-5:10	Emetine	−0.043	6.30E-05	1.22E-02	1.73	−7.25
CPC008_A375_6H:BRD-U88878891-000-01-9:10	BRD-U88878891	−0.043	3.61E-04	3.91E-02	1.74	−5.98
CPC014_HT29_6H:BRD-A26002865-001-01-5:10	Verrucarin-a	−0.043	2.88E-04	3.38E-02	1.71	−6.06
CPC017_MCF7_6H:BRD-K60511616-236-01-4:10	Pravastatin	−0.043	2.80E-04	3.35E-02	1.65	−5.84
CPC007_HT29_24H:BRD-K03067624-003-19-3:10	Emetine	−0.043	5.00E-04	4.75E-02	1.76	−5.82
CPC010_A375_6H:BRD-A24643465-001-05-3:10	Homoharringtonine	−0.043	2.03E-04	2.79E-02	1.76	−6.48
CPC015_MCF7_6H:BRD-K63550407-001-08-5:10	Erythromycin	−0.043	8.50E-05	1.47E-02	1.71	−6.96
CPC004_MCF7_6H:BRD-A25687296-300-03-5:10	Emetine	−0.043	8.78E-05	1.49E-02	1.87	−7.6
CPC008_MCF7_24H:BRD-K64409586-001-04-5:10	KU-C104488	−0.043	3.13E-05	7.79E-03	1.82	−8.19
CPC006_PC3_24H:BRD-A75517195-001-01-3:40	Thiazolopyrimidine	−0.043	2.03E-04	2.79E-02	1.79	−6.61
CPC006_LOVO_6H:BRD-K01976263-003-04-5:0.63	Emetine	−0.043	1.55E-04	2.30E-02	1.79	−6.81
CPC014_SKB_24H:BRD-K80622725-001-10-2:10	STK-397047	−0.043	2.50E-04	3.13E-02	1.7	−6.12
CPC012_MCF7_24H:BRD-K48935217-001-01-3:10	Epothilone	−0.0411	3.41E-04	3.77E-02	1.73	−5.99
CPC006_MCF7_24H:BRD-K01976263-003-04-5:0.63	Emetine	−0.0411	4.29E-04	4.34E-02	1.77	−5.96
CPC014_PC3_6H:BRD-K70549064-001-03-3:10	Staurosporine	−0.0411	4.54E-04	4.46E-02	1.69	−5.64
CPC008_A375_24H:BRD-K14749055-001-01-3:10	BRD-K14749055	−0.0411	6.13E-04	5.37E-02	1.76	−5.65
CPC017_A375_6H:BRD-A25687296-300-03-5:10	Emetine	−0.0411	2.18E-04	2.91E-02	1.72	−6.3
CPC002_HCC515_6H:BRD-K80348542-001-01-4:10	Cephaeline	−0.0411	8.58E-05	1.47E-02	1.9	−7.72
CPC019_HT29_6H:BRD-A70311631-001-05-9:10	BRD-A70311631	−0.0411	1.06E-03	7.60E-02	1.64	−4.87
LJP001_SKBR3_6H:BRD-K99252563-001-01-1:2	QL-XII-47	−0.0411	3.13E-04	3.56E-02	1.63	−5.7
LJP001_SKBR3_6H:BRD-K04923131-001-10-5:10	GSK-3-inhibitor-IX	−0.0411	3.94E-04	4.12E-02	1.61	−5.48
CPC014_HEPG2_6H:BRD-K83794624-001-01-7:10	Pirarubicin	−0.0411	7.99E-04	6.43E-02	1.67	−5.18
CPC006_SW948_6H:BRD-K05649647-001-03-7:20	BRD-K05649647	−0.0411	1.70E-04	2.45E-02	1.81	−6.83
CPC010_HEPG2_6H:BRD-A24643465-001-05-3:10	Homoharringtonine	−0.0411	5.07E-04	4.77E-02	1.72	−5.68
CPC013_SKB_24H:BRD-A14178283-001-01-1:10	BRD-A14178283	−0.0411	6.74E-05	1.26E-02	1.77	−7.39
MUC.CP004_MCF7_24H:BRD-K09638361-001-01-4:3.3333	SA-63133	−0.0411	3.13E-04	3.56E-02	1.61	−5.64
CPC013_VCAP_6H:BRD-A81530502-001-01-6:10	BRD-A81530502	−0.0393	9.82E-04	7.25E-02	1.71	−5.13
CPC006_HCC515_6H:BRD-K14696368-001-01-8:10	9-methyl-5H-6-thia-4,5-diaza-chrysene-6,6-dioxide	−0.0393	2.90E-04	3.38E-02	1.85	−6.54

**TABLE 9:** The top 50 drugs or natural products that reverse DEGs of astrocytes from entorhinal cortex in AD (AD00205 (disease) vs AD00201 (control) based on L1000FWD analysis.

Signature ID	Drug	Similarity score	*p*-value	q-value	Z-score	Combined score
CPC003_PC3_6H:BRD-K76534306-001-11-0:10	Enrofloxacin	−0.1071	7.61E-10	1.09E-05	1.87	−17.05
CPC006_U937_6H:BRD-K78126613-001-16-0:10	Menadione	−0.0893	5.38E-07	1.62E-03	1.78	−11.15
CPC019_HA1E_6H:BRD-K98824517-001-06-4:10	BRD-K98824517	−0.0893	3.48E-07	1.43E-03	1.67	−10.8
CPC018_MCF7_24H:BRD-K36055864-001-09-3:10	Cycloheximide	−0.0893	3.68E-07	1.43E-03	1.71	−10.97
CPC014_MCF7_24H:BRD-K16485616-001-03-0:10	Mocetinostat	−0.0893	6.83E-07	1.62E-03	1.73	−10.64
CPC016_A375_6H:BRD-K63516691-003-01-2:10	T-0156	−0.0833	2.78E-06	4.25E-03	1.7	−9.44
CPD001_MCF7_24H:BRD-K21680192-300-11-0:10	Mitoxantrone	−0.0833	1.51E-06	2.93E-03	1.67	−9.71
CPC016_MCF7_24H:BRD-K80348542-001-01-4:10	Cephaeline	−0.0833	2.64E-06	4.19E-03	1.68	−9.39
CPC017_HEPG2_6H:BRD-K04546108-066-01-5:10	JAK3-inhibitor-VI	−0.0833	1.18E-06	2.67E-03	1.72	−10.21
CPC013_A375_6H:BRD-K35638681-001-01-5:10	BRD-K35638681	−0.0833	2.27E-06	3.90E-03	1.74	−9.82
CPC014_PC3_24H:BRD-K95901403-001-01-1:10	XL-147	−0.0774	2.79E-05	1.44E-02	1.68	−7.66
CPC013_HCC515_6H:BRD-K94493764-001-01-3:10	BRD-K94493764	−0.0774	1.12E-05	9.40E-03	1.72	−8.53
CPC006_MCF7_6H:BRD-A67788537-001-01-7:120	Salermide	−0.0774	8.02E-06	8.18E-03	1.82	−9.25
CPD002_MCF7_6H:BRD-K42635745-001-19-8:10	Suloctidil	−0.0774	6.60E-06	7.63E-03	1.7	−8.79
CPC006_A375_6H:BRD-K05402890-001-02-7:0.35	BRD-K05402890	−0.0774	1.07E-05	9.40E-03	1.81	−9.01
CPC018_A549_6H:BRD-A71459254-001-02-8:10	Cymarin	−0.0774	1.09E-05	9.40E-03	1.69	−8.4
CPC010_A549_6H:BRD-K28916077-001-04-0:10	BRD-K28916077	−0.0714	4.69E-05	1.82E-02	1.76	−7.64
CPC014_PC3_24H:BRD-A18497,530-001-05-3:10	5-iodotubercidin	−0.0714	4.03E-05	1.79E-02	1.73	−7.62
LJP002_BT20_6H:BRD-A24396574-001-02-3:10	Celastrol	−0.0714	4.21E-05	1.79E-02	1.66	−7.25
CPC006_HT29_24H:BRD-K19894101-001-01-6:11.1	MST-312	−0.0714	4.79E-05	1.83E-02	1.8	−7.77
CPC018_HT29_6H:BRD-A80383043-001-01-7:10	BRD-A80383043	−0.0714	4.99E-05	1.87E-02	1.66	−7.13
CPC010_HEPG2_6H:BRD-K28916077-001-04-0:10	BRD-K28916077	−0.0714	2.41E-05	1.37E-02	1.8	−8.32
CPC004_PC3_6H:BRD-A09472452-015-11-9:10	Flecainide	−0.0714	3.16E-05	1.56E-02	1.84	−8.28
CPC019_A375_6H:BRD-K98824517-001-06-4:10	BRD-K98824517	−0.0714	2.64E-05	1.44E-02	1.7	−7.8
BRAF001_A375_24H:BRD-K16478699-001-05-0:0.625	PLX-4720	−0.0714	4.30E-05	1.79E-02	1.84	−8.05
CPC001_HA1E_6H:BRD-K02590140-001-01-2:10	O-2050	−0.0714	4.39E-05	1.79E-02	1.85	−
LJP001_MCF7_6H:BRD-K99252563-001-01-1:10	QL-XII-47	−-0.0714	5.90E-05	1.90E-02	1.62	−6.87
CPC012_MCF7_24H:BRD-K08307026-001-01-4:10	BRD-K08307026	−0.0714	4.49E-05	1.81E-02	1.76	−7.67
NMH001_NPC_24H:BRD-K14282469-001-09-8:10	LY-165163	−0.0714	5.10E-05	1.88E-02	1.61	−6.92
CPC010_HCC515_6H:BRD-K78385490-019-02-2:10	BRD-K78385490	−0.0714	1.01E-04	2.54E-02	1.73	−6.91
CPC006_MCF7_24H:BRD-K28360340-001-01-8:10	TW-37	−0.0714	5.54E-05	1.88E-02	1.81	−7.69
CPC008_A549_6H:BRD-K32944375-019-01-3:10	BRD-K32944375	−0.0714	8.47E-05	2.29E-02	1.75	−7.13
CPC012_PC3_6H:BRD-K28610502-001-01-0:10	RAN-05	−0.0714	1.36E-04	3.22E-02	1.7	−6.56
CPC009_A549_6H:BRD-K95138506-019-01-8:10	BRD-K95138506	−-0.0714	4.30E-05	1.79E-02	1.78	−7.77
CVD001_HUH7_6H:BRD-K76674262-001-01-7:2.5	Homoharringtonine	−0.0714	6.94E-05	2.07E-02	1.65	-6.85
CPC013_MCF7_6H:BRD-K35638681-001-01-5:10	BRD-K35638681	−0.0714	1.03E-04	2.57E-02	1.69	−6.73
CPC010_PC3_6H:BRD-K69676861-001-02-4:10	BRD-K69676861	−0.0714	4.39E-05	1.79E-02	1.76	−7.67
CPC015_A375_6H:BRD-K15409150-001-01-7:10	Penfluridol	−0.0655	3.01E-04	4.42E-02	1.69	−5.94
CPC014_PC3_6H:BRD-U86922168-000-01-3:10	QL-XII-47	−0.0655	1.65E-04	3.56E-02	1.73	−6.53
CPC002_HA1E_6H:BRD-K91370081-001-10-3:10	Anisomycin	−0.0655	1.62E-04	3.53E-02	1.85	−7.02
CPC006_TYKNU_6H:BRD-K92317137-001-04-0:10	BRD-K92317137	−0.0655	2.60E-04	4.26E-02	1.77	−6.36
CPC008_VCAP_24H:BRD-K44432556-001-03-0:10	VU-0418946-1	−0.0655	2.55E-04	4.26E-02	1.77	−6.36
CPC006_MCF7_6H:BRD-A62025033-001-01-8:10	Temsirolimus	−0.0655	3.59E-04	4.85E-02	1.77	−6.1
CPC006_HCC515_24H:BRD-K04430056-001-09-4:80	7-nitroindazole	−0.0655	1.43E-04	3.28E-02	1.84	−7.07
CPC014_A375_6H:BRD-U33728988-000-01-6:10	QL-X-138	−0.0655	2.85E-04	4.34E-02	1.69	−6.01
HOG001_MCF7_24H:BRD-K06854232-001-03-3:0.0045	AM-580	−0.0655	3.53E-04	4.80E-02	1.62	−5.58
CPC014_HT29_6H:BRD-K50720187-050-04-1:10	Flupirtine	−0.0655	1.55E-04	3.44E-02	1.71	−6.53
CPC019_VCAP_24H:BRD-K20000640-001-01-5:10	SA-247384	−0.0655	3.17E-04	4.59E-02	1.66	−5.82
CPC019_HT29_6H:BRD-K86027709-001-01-7:10	BRD-K86027709	−0.0655	2.95E-04	4.42E-02	1.65	−5.82
CPC006_SW620_6H:BRD-K06792661-001-01-9:10	Narciclasine	−0.0655	2.24E-04	4.00E-02	1.78	−6.49

**TABLE 10:** The top 50 drugs or natural products that reverse DEGs of astrocytes from entorhinal cortex in AD (AD00206 (disease) vs AD00201 (control) based on L1000FWD analysis.

Signature ID	Drug	Similarity score	*p*-value	q-value	Z-score	Combined score
CPC006_A375_24H:BRD-A75817871-001-04-2:40	Blebbistatin	−0.0593	1.31E-07	1.12E-03	1.79	−12.29
CPC004_MCF7_6H:BRD-K37991163-003-06-8:10	Paroxetine	−0.0571	1.12E-06	3.67E-03	1.79	−10.68
CPC014_HT29_6H:BRD-K53561341-001-02-6:10	KIN001-220	−0.0549	2.43E-06	4.92E-03	1.69	−9.5
CPC014_VCAP_24H:BRD-A52886023-001-01-7:10	Antimycin-a	−0.0527	4.90E-06	7.23E-03	1.69	−8.97
CPC009_VCAP_24H:BRD-K94390040-019-01-9:10	BRD-K94390040	−0.0527	7.95E-06	8.74E-03	1.75	−8.93
LJP001_SKBR3_24H:BRD-K19540840-001-04-5:10	Saracatinib	−0.0505	5.57E-06	7.94E-03	1.67	−8.77
CPC015_HEPG2_6H:BRD-K92093830-003-05-0:10	Doxorubicin	−0.0505	1.21E-05	1.19E-02	1.71	−8.42
CPC006_JHUEM2_6H:BRD-K12502280-001-01-5:11.1	TG-101348	−-0.0505	5.98E-06	8.08E-03	1.84	−9.63
CPC006_LOVO_6H:BRD-A62182663-001-01-4:10	YK-4279	−0.0484	2.82E-05	2.11E-02	1.8	−8.21
CPC003_HA1E_24H:BRD-K72783841-001-01-0:10	Tyrphostin-AG-555	−0.0484	4.68E-05	2.78E-02	1.82	−7.9
CPC006_A375_6H:BRD-K13049116-001-01-6:10	BMS-754807	−0.0484	3.53E-05	2.47E-02	1.82	−8.1
PCLB003_A375_24H:BRD-K95309561-001-19-7:0.12	Dienestrol	−0.0484	2.64E-05	2.02E-02	1.64	−7.5
CPC013_HEPG2_6H:BRD-K00954209-001-01-0:10	BRD-K00954209	−0.0484	1.04E-04	4.04E-02	1.71	−6.82
CPC013_VCAP_6H:BRD-A81530502-001-01-6:10	BRD-A81530502	−0.0484	4.39E-05	2.77E-02	1.72	−7.47
CPC018_A375_6H:BRD-K18787,491-001-07-8:10	U-0126	−0.0462	4.13E-05	2.77E-02	1.71	−7.5
CPC011_PC3_6H:BRD-K92093830-003-23-3:10	Doxorubicin	−0.0462	6.26E-05	3.18E-02	1.77	−7.46
CPC003_HA1E_24H:BRD-K17415526-001-02-7:10	Tyrphostin-AG-835	−0.0462	2.64E-04	6.89E-02	1.81	−6.46
CPC008_HEPG2_6H:BRD-K54687541-001-01-8:10	BRD-K54687541	−0.0462	6.26E-05	3.18E-02	1.8	−7.57
CPC006_HA1E_24H:BRD-K28360340-001-01-8:10	TW-37	−0.0462	1.66E-04	4.87E-02	1.79	−6.76
CPC014_SKB_24H:BRD-K89014967-001-01-9:10	AS-703026	−0.0462	1.48E-04	4.79E-02	1.7	−6.51
CPC018_A375_6H:BRD-K12244279-001-02-5:10	MEK1-2-inhibitor	−0.0462	6.46E-05	3.18E-02	1.7	−7.11
LJP001_SKBR3_24H:BRD-K49328571-001-06-9:2	Dasatinib	−0.0462	2.86E-04	7.25E-02	1.6	−5.67
CPC006_VCAP_6H:BRD-K12994359-001-07-7:177.6	Valdecoxib	−0.0462	9.89E-05	3.92E-02	1.78	−7.15
CPC014_HCC515_6H:BRD-M16762496-001-01-9:10	PIK-75	−0.0462	1.62E-04	4.87E-02	1.71	−6.47
LJP001_SKBR3_24H:BRD-K49328571-001-06-9:10	Dasatinib	−0.0462	2.50E-04	6.68E-02	1.62	−5.85
HOG002_A549_6H:BRD-K34581968-001-01-2:11.1	BMS-536924	−0.0462	1.32E-04	4.61E-02	1.65	−6.38
CPC013_SKB_24H:BRD-K49328571-001-04-4:10	Dasatinib	−0.0462	1.86E-04	5.27E-02	1.71	−6.38
CPC014_SKB_24H:BRD-K05804044-001-01-1:10	AZ-628	−0.0462	1.11E-04	4.14E-02	1.72	−-6.8
CPC012_MCF7_24H:BRD-K45842176-001-01-3:10	BRD-K45842176	−0.0462	1.62E-04	4.87E-02	1.73	−6.55
CPC014_MCF7_6H:BRD-K73293050-001-01-5:10	WZ-3146	−0.044	2.58E-04	6.78E-02	1.71	−6.14
CPC012_PC3_6H:BRD-A19248578-001-03-7:10	Latrunculin-b	−0.044	1.22E-04	4.37E-02	1.78	−6.97
CPC018_HEPG2_6H:BRD-K15588452-003-01-9:10	R-96544	−0.044	2.24E-04	6.12E-02	1.69	−6.15
CPC006_HA1E_24H:BRD-K68336408-001-04-2:56.78	Tyrphostin-AG-1478	−0.044	6.76E-04	1.10E-01	1.78	−5.64
CPC019_HT29_6H:BRD-K65366129-001-04-0:10	SD-6-035-A3	−0.044	1.25E-04	4.39E-02	1.69	−6.59
CPC014_HT29_6H:BRD-K16478699-001-02-7:10	PLX-4720	−0.044	2.24E-04	6.12E-02	1.71	−6.24
CPC006_A549_6H:BRD-K20285085-001-01-4:10	Fostamatinib	−0.044	9.80E-05	3.92E-02	1.83	−7.33
LJP002_MCF10A_6H:BRD-K41859756-001-03-5:0.4	NVP-AUY922	−0.044	1.69E-04	4.91E-02	1.65	−6.23
CPC018_HEPG2_6H:BRD-K46419649-001-01-8:10	U0126	−0.044	1.37E-04	4.69E-02	1.7	−6.57
CPC013_MCF7_24H:BRD-K16541732-001-01-3:10	BRD-K16541732	−0.044	9.90E-04	1.32E-01	1.67	−5.03
CPC006_A375_24H:BRD-K10705233-003-02-8:40	GW-405833	−0.044	8.40E-04	1.22E-01	1.76	−5.41
CPC012_SKB_24H:BRD-K08307026-001-01-4:10	BRD-K08307026	−0.044	2.51E-04	6.68E-02	1.72	−6.21
CPC012_MCF7_24H:BRD-K41220170-236-01-4:10	BRD-K41220170	−0.044	4.08E-04	8.32E-02	1.75	−5.94
MUC.CP004_MCF7_6H:BRD-K36627727-001-01-3:1.1111	Tamibarotene	−0.0418	4.72E-04	9.18E-02	1.62	−5.38
CPC006_HA1E_6H:BRD-K64634304-001-01-5:40	Tretinoin	−0.0418	7.53E-04	1.17E-01	1.8	−5.61
CPC003_HA1E_24H:BRD-K37691127-001-02-2:10	Hinokitiol	−0.0418	4.35E-04	8.70E-02	1.89	−6.34
CPC010_VCAP_6H:BRD-A04327189-001-11-0:10	Synephrine	−0.0418	3.12E-04	7.52E-02	1.81	−6.33
CPC018_NPC_24H:BRD-K22385716-001-01-7:10	LY-303511	−0.0418	6.46E-04	1.09E-01	1.66	−5.31
CPC003_PC3_24H:BRD-K17415526-001-02-7:10	Tyrphostin-AG-835	−0.0418	5.39E-04	9.88E-02	1.84	−6.02
CPC006_SW620_6H:BRD-K34581968-001-01-2:11.1	BMS-536924	−0.0418	1.25E-03	1.51E-01	1.77	−5.14
CPC019_PC3_24H:BRD-K92817986-001-01-7:10	BJM-CSC-19	−0.0418	7.34E-04	1.15E-01	1.68	−5.28

The drugs or natural products that could potentially reverse the gene signatures of astrocytes in AD groups (AD00203, AD00205, and AD00206) based on the L1000CDS^2^ web tool are provided in Supplementary Tables S1–S3, respectively. The L1000CDS^2^ analysis uncovered the natural products emetine, narciclasine, trichostatin A, homoharringtonine, ouabain, bufalin, and withaferin A, as well as synthetic drugs, such as dasatinib, that have the potential to reverse AD-associated gene signatures in astrocytes from AD patients.

## 4 Discussion

AD is a neurodegenerative disease of the brain and a major cause of the development of cognitive decline and dementia in the elderly (Winblad et al., 2016; Matthews et al., 2019). ADRD contributes to the majority of dementia cases worldwide (Winblad et al., 2016). Recent advances in genome sequencing technologies such as scRNA-Seq and snRNASeq are critical for deciphering the roles of heterogeneous cell populations in the brain at the single-cell level, and subsequent dissecting of these datasets using high throughput knowledge discovery platforms may provide clues as to why a particular group of cells is susceptible to AD and ADRD (Jiang et al., 2020; Wu and Zhang, 2020; Wang et al., 2021). Here, snRNASeq datasets of astrocytes isolated from the entorhinal cortex region of AD patients and healthy brains were obtained and analyzed using scREAD web-tool. scREAD includes 73 datasets from 16 studies, 10 brain regions, and 713,640 cells, and provides cell type and sub-cluster predictions, decipherment of DEGs, and discovery of cell type-specific regulons (Jiang et al., 2020; Wu and Zhang, 2020; Wang et al., 2021).

We observed that Wnt signaling and PI3K/AKT signaling pathways were dysregulated or impaired in astrocytes from the entorhinal cortex of AD patients. Wnt signaling is very important at the synapse and necessary for synaptic plasticity and maintenance in the brain (Palomer et al., 2019). The PI3K/AKT pathway regulates apoptosis, cell proliferation, and metabolism and is essential for protection against amyloid protein (Aβ)-induced neurotoxicity (Long et al., 2021). Neuroactive ligand-receptor interaction, axon guidance, Alzheimer’s disease, GABAergic synapse, glutamatergic synapse, etc. were negatively enriched or dysregulated in astrocytes from AD patients. GABAergic transmission is essential for all central nervous system functions (Luscher et al., 2011) and the GABAergic synapse pathway is impaired in the astrocytes of AD. This was also confirmed by GSEA analysis, which showed that the sets of genes involved in cellular components such as postsynaptic membrane, synaptic membrane, postsynapse, transporter complex, and interaction between neuroactive ligands and receptors were negatively enriched in the astrocytes of AD patients. Similarly, genes associated with glutamate receptor activity, neurotransmitter receptor activity, glutamate receptor signaling, heterophilic cell-cell adhesion via plasma membrane cell adhesion molecules, cell-cell adhesion via plasma membrane adhesion molecules, and behavior were also negatively enriched in the astrocytes of AD patients. Importantly, the downregulated GWAS genes in astrocytes derived from the entorhinal cortex, such as NKAIN3, LRRC4C, CADM2, DLC1, APOE, TNIK, GADD45G, FRMD4A, CTNNA2, NPAS3, NCKAP5, RORA, etc., associated with AD pathogenesis, can be used either as biomarkers for neuropathology, AD or LOAD (Riaz et al., 2021). Interestingly, we found signaling pathways associated with *Salmonella* infection, HPV infection, and human T-cell leukemia virus infection in the astrocytes of severe AD groups. Previous studies have shown that infections with *Salmonella* (Himmelhoch et al., 1947), HPV (Lin et al., 2020), and human T-cell leukemia virus (Lycke et al., 1993) are associated with dementia and cognitive decline in humans.

We have previously shown that natural products such as albiziasaponin-A, iso-orientin, and salvadorin can ameliorate the pathologies associated with AD *in vivo* (Rasool et al., 2018) and that the natural products could be useful for the treatment of age-related degenerative diseases (Kalamegam et al., 2020). In addition, we have recently shown that NGKD platforms can be successfully used to find drugs and natural products that may reverse disease-specific gene signatures (Pushparaj et al., 2021). Therefore, NGKD platforms can be used to find drugs and natural products that can potentially reverse AD-associated gene signatures in astrocytes. Here, we used iPathwayGuide, L1000FWD, and L1000CDS2 tools to identify promising drug-responsive molecules for ADRD. Comparative analysis of AD datasets using iPathwayGuide showed that antirheumatic drugs have insufficient signaling in astrocytes from AD patients. Disease-modifying antirheumatic drugs (DMARDs) are used to treat patients with rheumatoid arthritis (Bahlas et al., 2019) and recent studies have found that patients with rheumatoid arthritis taking antirheumatic drugs have a lower risk of developing dementia (Judge et al., 2017; Huang et al., 2019). Our finding is consistent with these studies that antirheumatic drugs can reverse AD-associated gene signatures in astrocytes. Similarly, vitamin E gene signatures were absent in astrocytes from AD. The role of vitamin E in the treatment of AD remains a controversial topic to date (Browne et al., 2019) and our results provide some evidence for the importance of vitamin E in the treatment of AD and ADRD. A recent study found that emetine may have the potential to clear amyloid-beta plaques in AD (Ahmad et al., 2019). The isoquinoline alkaloids emetine and its desmethyl analog cepaheline have been predicted to be protective against cognitive decline and AD (Fernández-Martínez et al., 2020). Withaferin A is a steroidal lactone and a withanolide found in the medicinal plant Withania somnifera, and a number of studies have shown that it plays a neuroprotective role in AD (Das et al., 2021). Narciclasine is an active constituent of the Lycoris radiata (L'Her.) herb. It is used in traditional Chinese medicine for the treatment of AD (Shen et al., 2019). A recent study found that senolytic therapy with a combination of dasatinib and quercetin reduced Aβ-associated oligodendrocyte progenitor cell senescence and cognitive decline in an AD model (Zhang et al., 2019). The histone deacetylase inhibitor trichostatin A (Hsing et al., 2015) increased albumin expression and Aβ clearance in APP/PS1 mice and improved cognitive deficits (Su et al., 2021). Trichostatin A increased the antioxidant capacity and cell viability of SH -SY5Y cells by enhancing Keap1-mediated inhibition of the Nrf2 pathway, thereby reducing amyloid-β peptide-mediated cell damage (Li et al., 2020). Importantly, we recently predicted the potential of withaferin A, narciclasine, and trichostatin A to reverse gene signatures in neuro- COVID (Pushparaj et al., 2021). However, the effects of natural products such as emetine, cephaeline, narciclasine, withaferin A, trichostatin A and drugs such as DMARDs and dasatinib which may be able to reverse AD gene signatures in astrocytes should be validated with appropriate experimental models from AD before being used for further clinical testing.

## 5 Conclusion

The present study provides a valuable method for analyzing snRNASeq datasets deposited in open-source repositories with NGKD platforms to decipher AD -specific pathways, genes, and drugs from synthetic and natural sources for the amelioration of AD-related disease pathologies such as ADRD. However, further studies are required to confirm these drugs and natural products that reverse the gene signatures of AD using appropriate experimental models to deduce the precise mechanisms of action, followed by appropriate clinical trials to evaluate the safety and efficacy of the likely therapeutic interventions for AD and ADRD in a typical clinical milieu. Our innovative approach of applying NGKD platforms to uncover AD-specific pathways and potential drugs and natural products that reverse the AD-specific signatures could be useful in the future for developing personalized medicine for AD patient care.

## Data Availability

The datasets presented in this study can be found in online repositories. The names of the repository/repositories and accession number(s) can be found in the article/Supplementary Material.

## Supplementary Material

Supplementary Figure S1 scREAD. A Single Cell RNA Sequencing Database for Alzheimer’s Disease (*Adapted from* *Jiang et al., 2020* *and shared under CC BY-NC-ND 4.0 license*)

Supplementary Figure S2 An example UMAP of healthy control and AD scREAD datasets. scREAD web tool was used to visualize all the cell types and sub-clusters of astrocytes in the entorhinal cortex region of the brain in AD and Healthy snRNASeq datasets using UMAP.

Supplementary Figure S3
**(A)** The iPathwayGuide analysis showing the differentially regulated genes in the Wnt signaling KEGG pathway in the astrocytes of the AD group (AD00203) compared to astrocytes of the healthy control group (AD00201). **(B)** Bar graph depicting the differentially regulated genes in the Wnt signaling pathway in the astrocytes of AD compared to healthy control.

Supplementary Figure S4
**(A)** The iPathwayGuide perturbation analysis based on the differentially regulated genes in the PI3K/AKT KEGG pathway in the astrocytes of the AD group (AD00203) compared to astrocytes of the healthy control group (AD00201). **(B)** Bar graph depicting the differentially regulated genes in the PI3K/AKT signaling pathway in the astrocytes of AD compared to healthy control.
