# Supplementary figures and images for: Decoding the Role of Astrocytes in the Entorhinal Cortex in Alzheimer’s Disease Using High-Dimensional Single-Nucleus RNA Sequencing Data and Next-Generation Knowledge Discovery Methodologies: Focus on Drugs and Natural Product Remedies for Dementia
Source: Front Pharmacol. 2022 Feb 28;12:720170. doi: 10.3389/fphar.2021.720170 (PMC8918735; doi:10.3389/fphar.2021.720170)

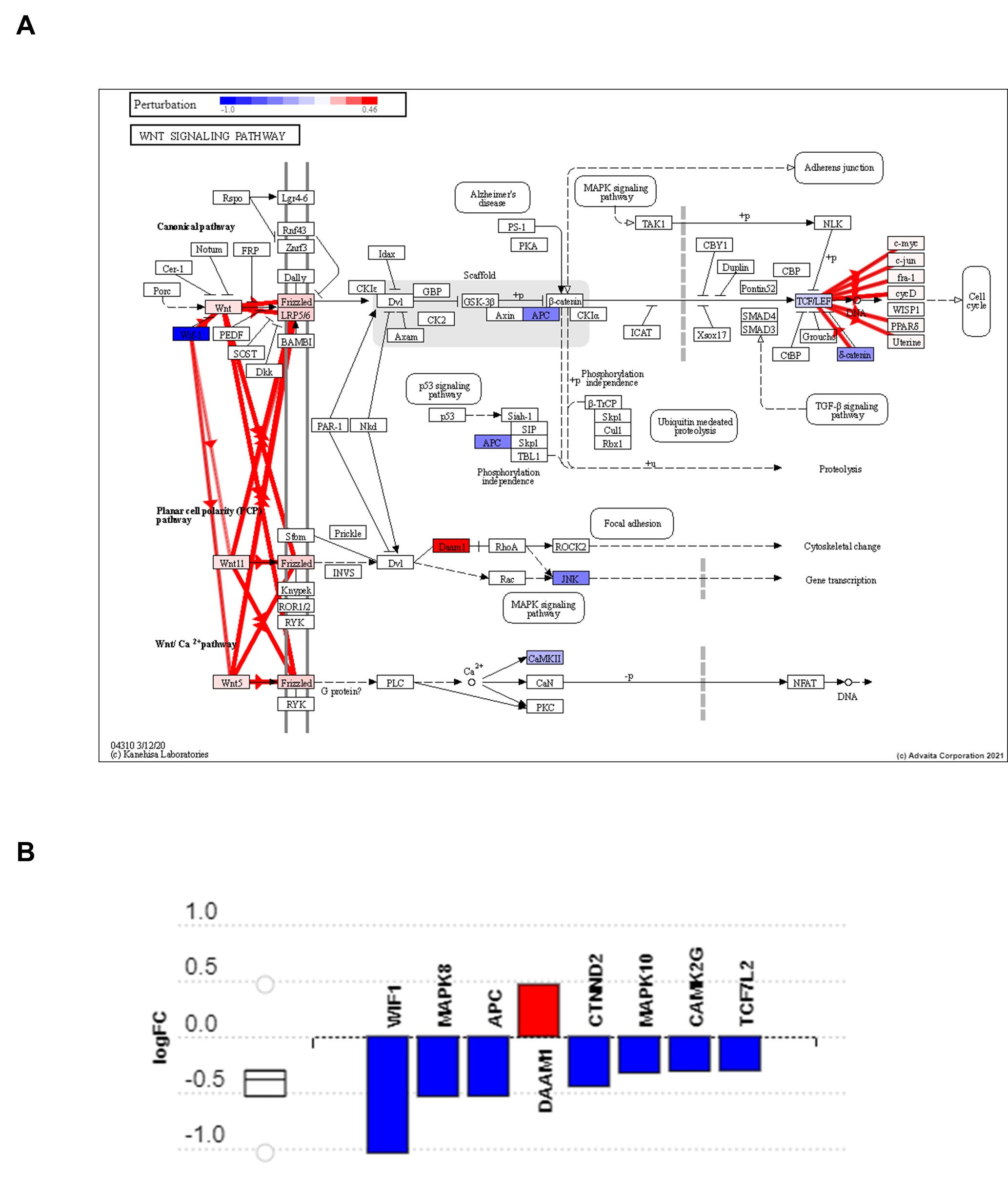

Supplement: Supplementary file 1 [file Image3.jpg]

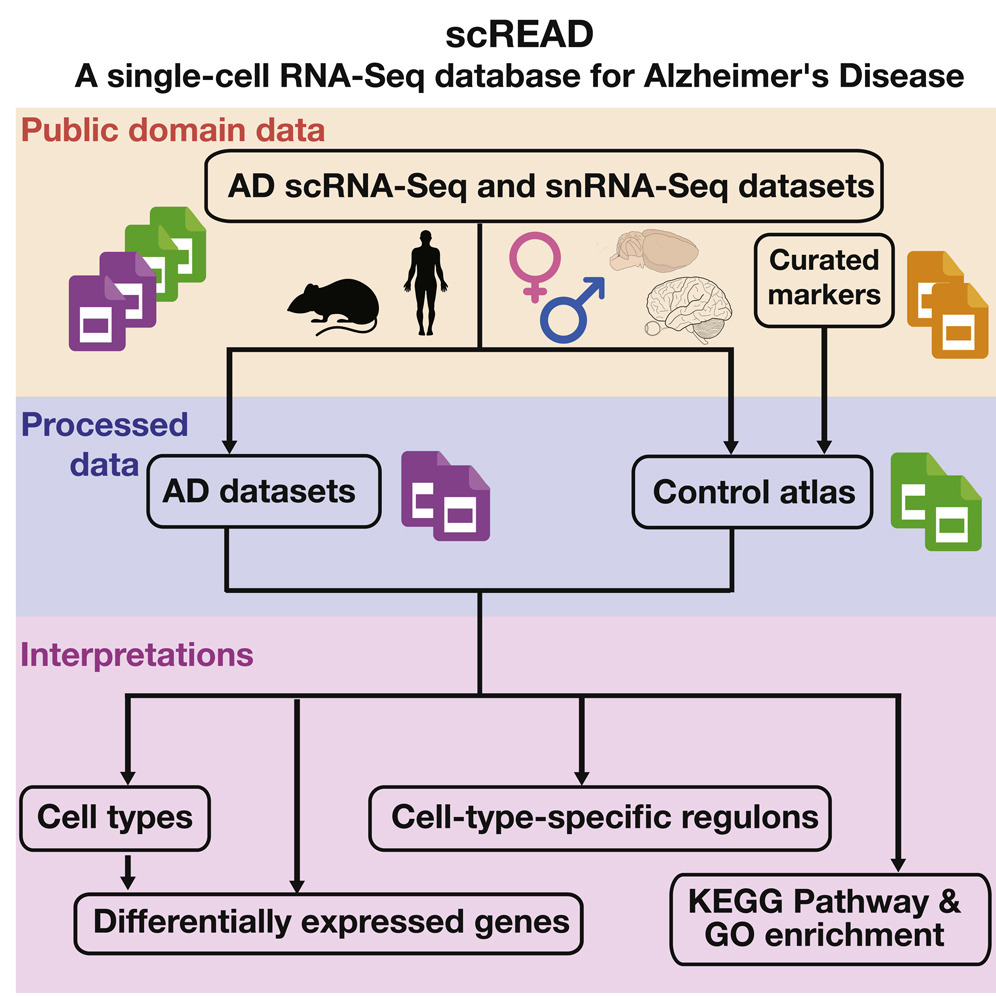

Supplement: Supplementary file 3 [file Image1.JPEG]

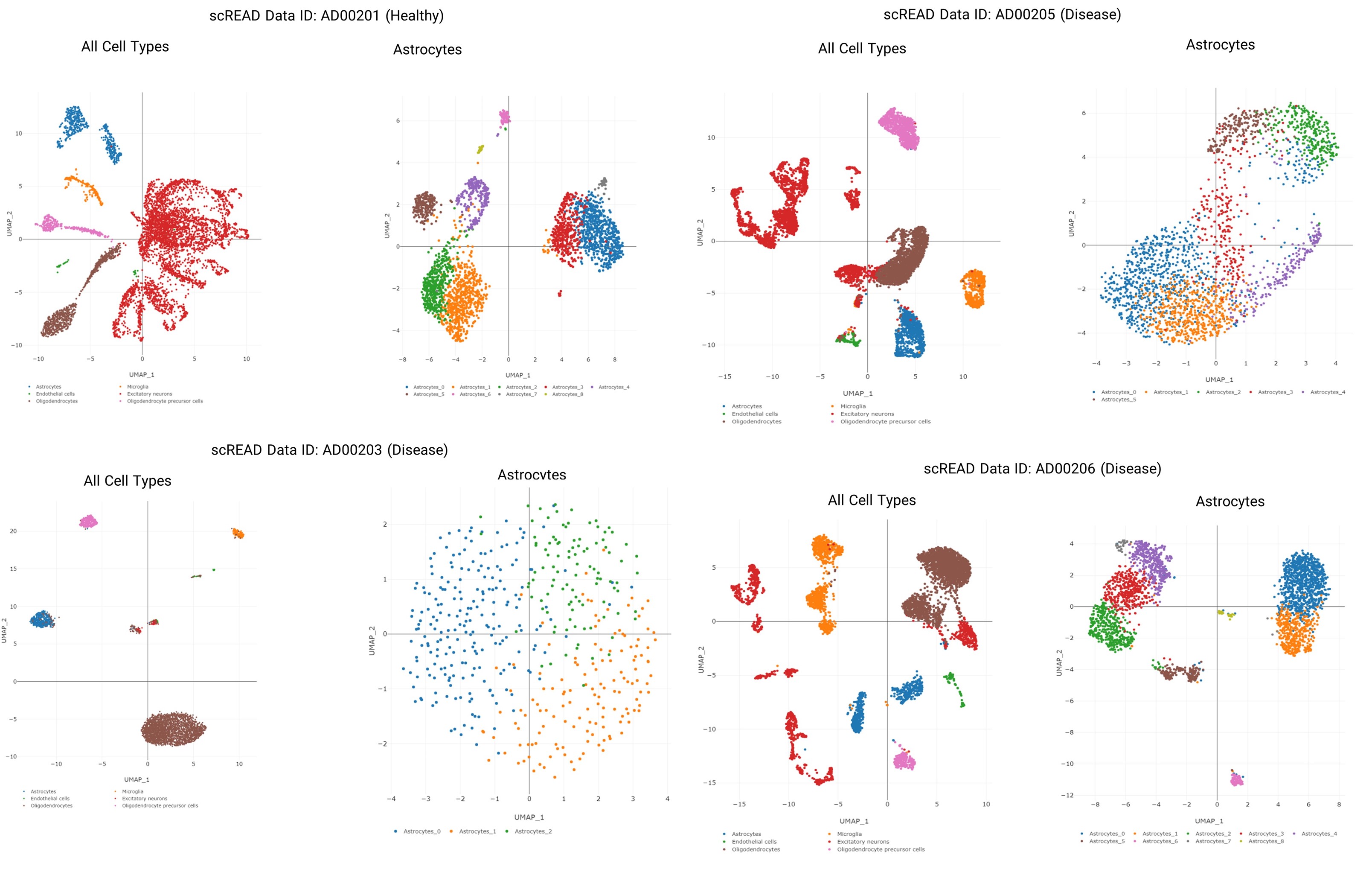

Supplement: Supplementary file 4 [file Image2.JPEG]

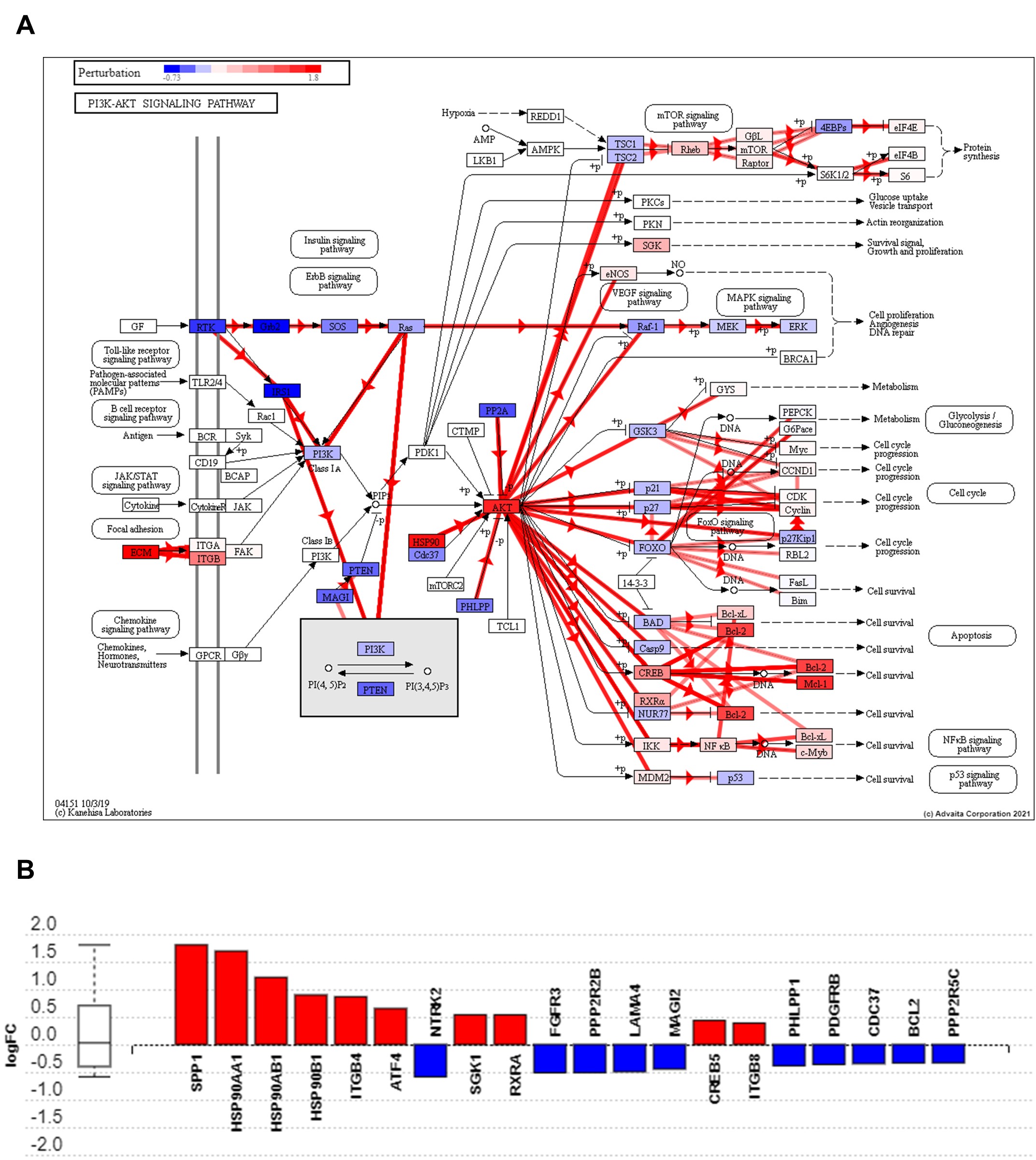

Supplement: Supplementary file 8 [file Image4.jpg]
