# Supplementary material for: Decoding the Role of Astrocytes in the Entorhinal Cortex in Alzheimer’s Disease Using High-Dimensional Single-Nucleus RNA Sequencing Data and Next-Generation Knowledge Discovery Methodologies: Focus on Drugs and Natural Product Remedies for Dementia
Source: Front Pharmacol. 2022 Feb 28;12:720170. doi: 10.3389/fphar.2021.720170 (PMC8918735; doi:10.3389/fphar.2021.720170)
Supplement: Supplementary file 2 [file Table1.docx]

**Supplementary Table 1.** The Top 50 drugs or natural products that reverse the DEGs of Astrocytes from Entorhinal Cortex in AD (AD00203 (Disease) vs AD00201 (Control) based on L1000CDS^2^ Analysis.

| Rank | score | Perturbation | Cell-line | Dose | Time |
| --- | --- | --- | --- | --- | --- |
| 1 | 0.0411 | EMETINE | MCF7 | 10.0um | 6.0h |
| 2 | 0.0411 | Narciclasine | WSUDLCL2 | 10.0um | 6.0h |
| 3 | 0.0411 | OTSSP167 | A375 | 0.37um | 24h |
| 4 | 0.0393 | Narciclasine | HA1E | 10.0um | 24.0h |
| 5 | 0.0393 | Emetine Dihydrochloride Hydrate (74) | MCF7 | 0.63um | 24.0h |
| 6 | 0.0374 | BRD-K56411643 | VCAP | 10.0um | 24.0h |
| 7 | 0.0374 | glycopyrrolate | MCF7 | 10.0um | 24.0h |
| 8 | 0.0374 | T5212475 | VCAP | 10.0um | 24.0h |
| 9 | 0.0374 | QL-XII-47 | HEPG2 | 1.11um | 24h |
| 10 | 0.0374 | CGP-60474 | A375 | 0.04um | 24h |
| 11 | 0.0355 | mitoxantrone | A375 | 10.0um | 24.0h |
| 12 | 0.0355 | Emetine Dihydrochloride Hydrate (74) | HT29 | 0.63um | 24.0h |
| 13 | 0.0355 | EMETINE HYDROCHLORIDE | A549 | 10.0um | 24.0h |
| 14 | 0.0355 | HY-10005 | ASC | 10.0um | 24.0h |
| 15 | 0.0355 | NP-004102 | SKB | 10.0um | 24.0h |
| 16 | 0.0355 | STK397047 | SKB | 10.0um | 24.0h |
| 17 | 0.0355 | LDN-193189 | HS578T | 10um | 24h |
| 18 | 0.0355 | CGP-60474 | A375 | 0.12um | 24h |
| 19 | 0.0336 | Cycloheximide | PC3 | 10.0um | 24.0h |
| 20 | 0.0336 | S1230 | VCAP | 10.0um | 24.0h |
| 21 | 0.0336 | BRD-K06543683 | ASC | 10.0um | 24.0h |
| 22 | 0.0318 | Homoharringtonine | HA1E | 10.0um | 24.0h |
| 23 | 0.0318 | OUABAIN | HA1E | 10.0um | 6.0h |
| 24 | 0.0318 | CYCLOHEXIMIDE | PC3 | 10.0um | 24.0h |
| 25 | 0.0318 | EMETINE HYDROCHLORIDE | HT29 | 10.0um | 6.0h |
| 26 | 0.0318 | BRD-K56653679 | SKB | 10.0um | 24.0h |
| 27 | 0.0318 | BRD-A84102390 | MCF7 | 10.0um | 24.0h |
| 28 | 0.0318 | EI-293 | ASC | 10.0um | 24.0h |
| 29 | 0.0318 | BRD-K92093830 | ASC | 10.0um | 24.0h |
| 30 | 0.0299 | BRD-K80348542 | HCC515 | 10.0um | 24.0h |
| 31 | 0.0299 | bufalin | HA1E | 10.0um | 24.0h |
| 32 | 0.0299 | EMETINE | HA1E | 10.0um | 6.0h |
| 33 | 0.0299 | Cycloheximide | HCC515 | 10.0um | 24.0h |
| 34 | 0.0299 | EMETINE | PC3 | 10.0um | 6.0h |
| 35 | 0.0299 | Emetine Dihydrochloride Hydrate (74) | HA1E | 0.63um | 24.0h |
| 36 | 0.0299 | T542500 | VCAP | 10.0um | 24.0h |
| 37 | 0.0299 | S1249 | ASC | 10.0um | 24.0h |
| 38 | 0.0299 | S1216 | MCF7 | 10.0um | 24.0h |
| 39 | 0.0299 | BRD-K38615104 | NPC | 10.0um | 24.0h |
| 40 | 0.0299 | BRD-K19220233 | SKB | 10.0um | 24.0h |
| 41 | 0.0299 | S1205 | SKB | 10.0um | 24.0h |
| 42 | 0.0299 | BRD-A68009927 | ASC | 10.0um | 24.0h |
| 43 | 0.0299 | BRD-A73909368 | ASC | 10.0um | 24.0h |
| 44 | 0.0299 | BRD-K43389675 | ASC | 10.0um | 24.0h |
| 45 | 0.0299 | chelerythrine chloride | HEPG2 | 10um | 24h |
| 46 | 0.0299 | alvocidib | SKBR3 | 0.12um | 24h |
| 47 | 0.028 | Anisomycin | HA1E | 10.0um | 24.0h |
| 48 | 0.028 | BRD-K80348542 | HA1E | 10.0um | 6.0h |
| 49 | 0.028 | EMETINE | HA1E | 10.0um | 24.0h |
| 50 | 0.028 | Ro 31-8220 mesylate | PC3 | 10.0um | 6.0h |
