# Supplementary material for: Decoding the Role of Astrocytes in the Entorhinal Cortex in Alzheimer’s Disease Using High-Dimensional Single-Nucleus RNA Sequencing Data and Next-Generation Knowledge Discovery Methodologies: Focus on Drugs and Natural Product Remedies for Dementia
Source: Front Pharmacol. 2022 Feb 28;12:720170. doi: 10.3389/fphar.2021.720170 (PMC8918735; doi:10.3389/fphar.2021.720170)
Supplement: Supplementary file 5 [file Table2.docx]

**Supplementary Table 2.** The Top 50 drugs or natural products that reverse the DEGs of Astrocytes from Entorhinal Cortex in AD (AD00205 (Disease) vs AD00201 (Control) based on L1000CDS2 Analysis.

| Rank | score | Perturbation | Cell-line | Dose | Time |
| --- | --- | --- | --- | --- | --- |
| 1 | 0.0774 | Homoharringtonine | PC3 | 10.0um | 6.0h |
| 2 | 0.0714 | EMETINE | HA1E | 10.0um | 6.0h |
| 3 | 0.0714 | gemcitabine | A375 | 0.08um | 24.0h |
| 4 | 0.0655 | Narciclasine | WSUDLCL2 | 10.0um | 6.0h |
| 5 | 0.0655 | BRD-U86922168 | PC3 | 10.0um | 6.0h |
| 6 | 0.0655 | AT-7519 | HA1E | 3.33um | 24h |
| 7 | 0.0595 | Digoxigenin | VCAP | 10.0um | 24.0h |
| 8 | 0.0595 | 528116.cdx | COV644 | 0.09um | 6.0h |
| 9 | 0.0595 | BRD-K04853698 | NCIH2073 | 10.0um | 6.0h |
| 10 | 0.0595 | NICLOSAMIDE | U937 | 10.0um | 6.0h |
| 11 | 0.0595 | Parthenolide | VCAP | 20.0um | 6.0h |
| 12 | 0.0595 | NCGC00238536-01 | HEPG2 | 10.0um | 6.0h |
| 13 | 0.0595 | V4877 | HA1E | 10.0um | 6.0h |
| 14 | 0.0595 | BRD-K80348542 | MCF7 | 10.0um | 24.0h |
| 15 | 0.0595 | BRD-K49010888 | MCF7 | 10.0um | 24.0h |
| 16 | 0.0595 | BRD-K53308430 | VCAP | 10.0um | 24.0h |
| 17 | 0.0595 | AZD-8330 | HT29 | 1.11um | 24h |
| 18 | 0.0595 | BMS-345541 | HA1E | 10um | 24h |
| 19 | 0.0536 | Danazol | HA1E | 10.0um | 6.0h |
| 20 | 0.0536 | manumycin A | A375 | 10.0um | 24.0h |
| 21 | 0.0536 | Narciclasine | HA1E | 10.0um | 6.0h |
| 22 | 0.0536 | COT-10b | HT115 | 44.4um | 6.0h |
| 23 | 0.0536 | Narciclasine | HT29 | 10.0um | 24.0h |
| 24 | 0.0536 | BRD-K92317137 | NOMO1 | 10.0um | 6.0h |
| 25 | 0.0536 | PERHEXILINE MALEATE | SNGM | 10.0um | 6.0h |
| 26 | 0.0536 | Narciclasine | TYKNU | 10.0um | 6.0h |
| 27 | 0.0536 | BRD-K17140735 | TYKNU | 11.1um | 6.0h |
| 28 | 0.0536 | PAC 1 | VCAP | 10.0um | 24.0h |
| 29 | 0.0536 | NCGC00183696-01 | VCAP | 10.0um | 24.0h |
| 30 | 0.0536 | BRD-K28916077 | HEPG2 | 10.0um | 6.0h |
| 31 | 0.0536 | vorinostat | MCF7 | 10.0um | 24.0h |
| 32 | 0.0536 | BRD-K76840893 | MCF7 | 10.0um | 24.0h |
| 33 | 0.0536 | BRD-A25687296 | MCF7 | 10.0um | 6.0h |
| 34 | 0.0536 | BRD-K98824517 | A375 | 10.0um | 6.0h |
| 35 | 0.0536 | BRD-K52522949 | MCF7 | 10.0um | 24.0h |
| 36 | 0.0536 | BRD-K06009608 | VCAP | 10.0um | 24.0h |
| 37 | 0.0536 | regorafenib | HEPG2 | 3.33um | 24h |
| 38 | 0.0536 | mitoxantrone | SKBR3 | 3.33um | 24h |
| 39 | 0.0536 | LDN-193189 | SKBR3 | 10um | 3h |
| 40 | 0.0536 | A443654 | SKBR3 | 0.37um | 24h |
| 41 | 0.0536 | QL-XII-47 | SKBR3 | 1.11um | 3h |
| 42 | 0.0476 | Parthenolide | VCAP | 10.0um | 24.0h |
| 43 | 0.0476 | OUABAIN | HA1E | 10.0um | 6.0h |
| 44 | 0.0476 | Dorsomorphin dihydrochloride | HA1E | 10.0um | 6.0h |
| 45 | 0.0476 | EMETINE | MCF7 | 10.0um | 6.0h |
| 46 | 0.0476 | NSC 663284 | VCAP | 10.0um | 24.0h |
| 47 | 0.0476 | Etoposide | A375 | 10.0um | 6.0h |
| 48 | 0.0476 | trichostatin A | HT29 | 10.0um | 24.0h |
| 49 | 0.0476 | BRD-K57080016 | A375 | 80.0um | 24.0h |
| 50 | 0.0476 | BRD-K92317137 | A375 | 10.0um | 6.0h |
