# Supplementary material for: Decoding the Role of Astrocytes in the Entorhinal Cortex in Alzheimer’s Disease Using High-Dimensional Single-Nucleus RNA Sequencing Data and Next-Generation Knowledge Discovery Methodologies: Focus on Drugs and Natural Product Remedies for Dementia
Source: Front Pharmacol. 2022 Feb 28;12:720170. doi: 10.3389/fphar.2021.720170 (PMC8918735; doi:10.3389/fphar.2021.720170)
Supplement: Supplementary file 7 [file Table3.docx]

**Supplementary Table 3.** The Top 50 drugs or natural products that reverse the DEGs of Astrocytes from Entorhinal Cortex in AD (AD00206 (Disease) vs AD00201 (Control) based on L1000CDS2 Analysis.

| Rank | score | Perturbation | Cell-line | Dose | Time |
| --- | --- | --- | --- | --- | --- |
| 1 | 0.0642 | PLX-4032 | A375 | 10.0um | 24.0h |
| 2 | 0.0612 | AZD-8330 | HT29 | 1.11um | 24h |
| 3 | 0.0581 | AS605240 | A375 | 10.0um | 24.0h |
| 4 | 0.0581 | alvocidib | HA1E | 0.12um | 24h |
| 5 | 0.055 | mitoxantrone | HA1E | 3.33um | 24h |
| 6 | 0.052 | AT-7519 | HA1E | 3.33um | 24h |
| 7 | 0.052 | CGP-60474 | HA1E | 3.33um | 24h |
| 8 | 0.0489 | A443654 | HA1E | 1.11um | 24h |
| 9 | 0.0489 | DCC-2036 | A375 | 3.33um | 24h |
| 10 | 0.0459 | trichostatin A | HA1E | 10.0um | 6.0h |
| 11 | 0.0459 | BRD-K57080016 | A375 | 80.0um | 24.0h |
| 12 | 0.0459 | PLX-4720 | A375 | 10um | 24h |
| 13 | 0.0459 | CGP-60474 | HA1E | 1.11um | 24h |
| 14 | 0.0459 | AZD-5438 | HA1E | 10um | 24h |
| 15 | 0.0459 | CGP-60474 | HA1E | 0.04um | 24h |
| 16 | 0.0459 | CGP-60474 | MCF10A | 0.37um | 24h |
| 17 | 0.0459 | PD-0325901 | A375 | 10um | 24h |
| 18 | 0.0428 | PP-110 | A375 | 22.2um | 24.0h |
| 19 | 0.0428 | BI 2536 | HT29 | 10.0um | 24.0h |
| 20 | 0.0428 | BRD-K92317137 | VCAP | 10.0um | 6.0h |
| 21 | 0.0428 | PLX-4720 | A375 | 1.11um | 24h |
| 22 | 0.0428 | AZD-8330 | A375 | 3.33um | 24h |
| 23 | 0.0428 | PLX-4720 | HT29 | 10um | 24h |
| 24 | 0.0428 | PD-0325901 | A375 | 1.11um | 24h |
| 25 | 0.0428 | PD-0325901 | A375 | 3.33um | 24h |
| 26 | 0.0428 | alvocidib | BT20 | 0.37um | 24h |
| 27 | 0.0428 | AT-7519 | HA1E | 10um | 24h |
| 28 | 0.0428 | PD-0325901 | A375 | 0.04um | 24h |
| 29 | 0.0428 | PD-0325901 | A375 | 0.12um | 24h |
| 30 | 0.0398 | trichostatin A | HT29 | 10.0um | 24.0h |
| 31 | 0.0398 | BMS-536924 | A375 | 11.1um | 24.0h |
| 32 | 0.0398 | vorinostat | PC3 | 10.0um | 24.0h |
| 33 | 0.0398 | KU 0060648 trihydrochloride | SKM1 | 10.0um | 6.0h |
| 34 | 0.0398 | PERHEXILINE MALEATE | SNGM | 10.0um | 6.0h |
| 35 | 0.0398 | TW 37 | VCAP | 10.0um | 24.0h |
| 36 | 0.0398 | BRD-K02562327 | VCAP | 10.0um | 24.0h |
| 37 | 0.0398 | PD-184352 | A375 | 0.12um | 24h |
| 38 | 0.0398 | PD-0325901 | A375 | 0.04um | 24h |
| 39 | 0.0398 | SB590885 | HT29 | 10um | 24h |
| 40 | 0.0398 | selumetinib | A375 | 0.37um | 24h |
| 41 | 0.0398 | alvocidib | HA1E | 10um | 24h |
| 42 | 0.0398 | withaferin-a | HA1E | 10um | 24h |
| 43 | 0.0398 | BMS-345541 | HA1E | 10um | 24h |
| 44 | 0.0398 | AZD-5438 | MCF10A | 10um | 24h |
| 45 | 0.0398 | dasatinib | MDAMB231 | 0.12um | 24h |
| 46 | 0.0398 | PHA-793887 | SKBR3 | 10um | 24h |
| 47 | 0.0398 | cabozantinib | A375 | 10um | 24h |
| 48 | 0.0398 | CGP-60474 | A375 | 0.12um | 24h |
| 49 | 0.0367 | trichostatin A | HA1E | 10.0um | 6.0h |
| 50 | 0.0367 | SB 218078 | HEPG2 | 10.0um | 6.0h |
